# Cucumber Possesses a Single Terminal Alternative Oxidase Gene That is Upregulated by Cold Stress and in the Mosaic (MSC) Mitochondrial Mutants

**DOI:** 10.1007/s11105-015-0883-9

**Published:** 2015-04-21

**Authors:** Tomasz L. Mróz, Michael J. Havey, Grzegorz Bartoszewski

**Affiliations:** Department of Plant Genetics, Breeding and Biotechnology, Faculty of Horticulture, Biotechnology and Landscape Architecture, Warsaw University of Life Sciences, ul. Nowoursynowska 159, 02-776 Warsaw, Poland; Agricultural Research Service, U.S. Department of Agriculture, Vegetable Crops Unit, Department of Horticulture, University of Wisconsin, 1575 Linden Dr., Madison, WI 53706 USA

**Keywords:** Alternative oxidase, *Cucumis sativus*, Gene structure, Mitochondrial mutants

## Abstract

Alternative oxidase (AOX) is a mitochondrial terminal oxidase which is responsible for an alternative route of electron transport in the respiratory chain. This nuclear-encoded enzyme is involved in a major path of survival under adverse conditions by transfer of electrons from ubiquinol instead of the main cytochrome pathway. AOX protects against unexpected inhibition of the cytochrome c oxidase pathway and plays an important role in stress tolerance. Two AOX subfamilies (AOX1 and AOX2) exist in higher plants and are usually encoded by small gene families. In this study, genome-wide searches and cloning were completed to identify and characterize AOX genes in cucumber (*Cucumis sativus* L.). Our results revealed that cucumber possesses no AOX1 gene(s) and only a single AOX2 gene located on chromosome 4. Expression studies showed that AOX2 in wild-type cucumber is constitutively expressed at low levels and is upregulated by cold stress. AOX2 transcripts and protein were detected in leaves and flowers of wild-type plants, with higher levels in the three independently derived mosaic (MSC) mitochondrial mutants. Because cucumber possesses a single AOX gene and its expression increases under cold stress and in the MSC mutants, this plant is a unique and intriguing model to study AOX expression and regulation particularly in the context of mitochondria-to-nucleus retrograde signaling.

**Electronic supplementary material:**

The online version of this article (doi:10.1007/s11105-015-0883-9) contains supplementary material, which is available to authorized users.

## Introduction

Alternative oxidase (AOX) is a nuclear-encoded ubiquinol—oxygen oxidoreductase associated with an alternative mitochondrial pathway that reduces the potential of reactive oxygen species (ROS) produced during respiratory electron transport (Elthon and McIntosh 1987; Vanlerberghe and McIntosh 1997; Maxwell et al. 1999). AOX is located on the matrix face of the inner mitochondrial membrane with binuclear iron atoms at the catalytic site (Siedow et al. 1995; Andersson and Nordlund 1999). The first AOX gene was cloned and characterized in the thermogenic plant *Sauromatum guttatum* (Schott) (Rhoads and McIntosh 1991). Later, AOX genes were described in some non-thermogenic plants (McDonald 2008). In higher plants, AOX is encoded by small gene families (three to five members), for example five genes in *Arabidopsis* (*Arabidopsis thaliana*) (Saisho et al. 1997; Clifton et al. 2005), four in tomato (*Solanum lycopersicum*) (Borecký et al. 2006), and three in maize (*Zea mays*) (Karpova et al. 2002) and soybean (*Glycine max*) (Whelan et al. 1996). Two AOX subfamilies (AOX1 and AOX2) can be distinguished by their sequences (Considine et al. 2002). Expansion and functional diversification of AOX1 genes have been observed in both monocots and eudicots, while the AOX2 subfamily so far has been found only in eudicots (Considine et al. 2002; Costa et al. 2014). In the Fabales, AOX2 gene duplications have been described (Cavalcanti et al. 2013; Costa et al. 2014). Recently, a detailed classification of AOX families based on protein sequences was proposed, which divides the AOX2 subfamily into two types: constitutively expressed AOX2a–c and stress-responsive AOX2d (Cavalcanti et al. 2013; Costa et al. 2014).

Each member of the AOX gene family has specific tissue and developmental stage expression patterns (Saisho et al. 1997). AOX1 genes are upregulated by ROS, salicylic acid (SA), and stresses (cold, drought, salinity, pathogens) (Considine et al. 2002; Clifton et al. 2005; Borecký et al. 2006; Feng et al. 2010; Zhang et al. 2012). Increased AOX expression lowers mitochondrial ROS production from an impaired respiratory chain (Maxwell et al. 1999). ROS also act as signaling molecules affecting retrograde (chloroplast or mitochondrial to nucleus) signaling (Apel and Hirt 2004; Woodson and Chory 2008; Leister 2012). In higher plants, one of the most common responses to the mitochondria-to-nucleus signaling is related to increased AOX level (Zarkovic et al. 2005; Rhoads and Subbaiah 2007). AOX is likely responsive to multiple and complex signals of respiratory status that are controlling nuclear gene expression including AOX gene (De Clercq et al. 2013; Vanlerberghe 2013). Recent studies indicate that NAC domain-containing protein 17 (ANAC017) is one of the direct positive regulators of *Aox1a* in *Arabidopsis* and contributes to the retrograde signaling network (Ng et al. 2013). Therefore, AOX is increasingly being studied in the context of retrograde regulation between the nucleus and mitochondria (Giraud et al. 2009; Ivanowa et al. 2014; Ng et al. 2014), as well as in the acclimation of plants to biotic and abiotic stresses (Giraud et al. 2008; McDonald 2008; Cvetkovska and Vanlerberghe 2013). Robson and Vanlerberghe (2002) reported that lack of AOX in transgenic tobacco cells dramatically influenced programmed cell death (PCD). Moreover, overexpression of AOX resulted in smaller hypersensitive response lesions, suggesting that AOX may act as a suppressor of PCD in virus-infected leaves (Ordog et al. 2002). Plant mitochondrial mutants or nuclear mutants which affect mitochondrial function show mutant-specific expression patterns of AOX genes (Karpova et al. 2002; Sosso et al. 2012).

Cucumber is a unique model plant to study organellar genetics (Havey et al. 2002; Bartoszewski et al. 2007). It is characterized by differential transmission of the three plant genomes (paternal for mitochondrial, maternal for plastids, and bi-parental for nuclear) (Havey 1997; Havey et al. 1998); relatively large mitochondrial DNA (mtDNA) in which inter- and intramolecular recombination results in mtDNA rearrangements (Lilly et al. 2001; Bartoszewski et al. 2004; Alverson et al. 2011); and availability of cell cultures developed mosaic (MSC) mitochondrial mutants possessing unique rearrangements within their mtDNA (Malepszy et al. 1996; Bartoszewski et al. 2007). Several MSC lines with distinct phenotypes have been derived independently in cell cultures from the highly inbred line B and it has been proposed that the passage through cell cultures may be used as a way to produce cucumber mitochondrial mutants (Ładyżyński et al. 2002; Bartoszewski et al. 2007). The most extensively studied MSC16 mutant has a large 15.1 kb deletion of a non-coding region (JLV5) and duplication of the RPL5 region in its mtDNA (Lilly et al. 2001; Bartoszewski et al. 2004), negatively affecting respiratory complex I and increasing ROS production (Juszczuk et al. 2007; Szal et al. 2009).

The aim of this study was genome-wide identification, cloning, and expression profiling of AOX genes in cucumber. Recently, Costa et al. (2014) suggested that Cucurbitales possesses only one AOX2. We provide direct experimental evidence that cucumber possesses no AOX1 gene(s) and only a single AOX2 gene on chromosome 4 encoding an AOX2a–c type of protein. Additionally, we show that cucumber AOX2 is transcriptionally upregulated in different MSC mutants and by cold treatment. Expression studies suggest a distinct role and complex adjustment of the cucumber AOX2 gene making cucumber, for which lines with different mtDNA rearrangements are available, a unique and intriguing plant model to investigate the regulation of the AOX expression and mitochondrial retrograde signaling.

## Materials and Methods

### Plant Material

Origins and seed sources of highly inbred line B, which in this study represents wild-type (interchangeably hereinafter referred to as the control line), and MSC mitochondrial mutant lines 3, 12, and 16 (each independently derived from line B) have been described (Malepszy et al. 1996; Bartoszewski et al. 2007).

### Tissue Collection from Field Grown Plants

Cucumber plants were grown in the experimental field of the Department of Horticulture, University of Wisconsin (Madison, WI, USA) during summer growth season under standard open-field cultivation procedure. Cucumber transplants were prepared in the greenhouse and planted at the end of May to the field. Leaves and male flowers were collected in the second part of June from 6- to 8-week-old plants (11–15 leaves), pooled (*n* = 12), and immediately deep-frozen in liquid nitrogen. The tissue was used in Northern and Western blot analysis.

### Plant Experiments Under Controlled Conditions

Seeds were surface-sterilized in a 10 % (*v*/*v*) solution of commercial bleach (0.45 % sodium hypochlorite final concentration) for 5 min, washed three times in distilled water, and allowed to imbibe in distilled water for 24 h at 28 °C. Imbibed seeds were sown into the commercial soil mixture Humovit brand (Hit-Torf, Łomża, Poland) and grown in CMP6050 phytotron (Conviron Winnipeg, Canada). The plants were grown at 25 °C during the day and 20 °C during the night and a 16-h photoperiod under fluorescent white light: 400 μmol m^−2^ s^−1^ and a relative humidity of 55–65 %. MSC mutants are characterized by slower growth (Malepszy et al. 1996; Bartoszewski et al. 2007); therefore, to compensate the physiological growth phases, MSC12 and MSC16 lines were sown 2 days earlier than the control line. Plant tissues were collected when the cucumber plants possessed a fully expanded first true leaf (about 12–14 days after sowing).

For cold treatment, cucumber plants possessing fully expanded first true leaf were chilled at 4 °C for 8 h under fluorescent white light 400 μmol m^−2^ s^−1^ (Kozik and Werner 2008). After cold treatment, the plants were placed back under the same temperature and light conditions as before chilling and were further cultivated. Plant tissues were collected before chilling, 1 h, 48 h (2 days), and 144 h (6 days) after chilling. In all experiments, tissue was collected by cutting the plants 0.5 cm below the cotyledons. Collected tissues for each line and experimental conditions were pooled (*n* = 5), immediately frozen in liquid nitrogen, ground using mortar and pestle, transferred to 1.5-ml tubes, and used for RNA or protein isolation and RT-qPCR and Western blot analyses.

### Cloning of AOX2 Gene from Cucumber

Genomic DNA from MSC16 was subjected to PCR to amplify AOX gene(s) using the degenerated oligonucleotides P1 and P2 (Table 1) designed for conservative regions of AOX proteins as described by Saisho et al. (1997). A single 446 bp fragment was amplified and cloned after TA tailing. DNA of 15 putative cucumber AOX clones were sequenced using Sanger method at the IBB Sequencing and Oligo Synthesis Core (IBB Oligo, Warsaw, Poland) to confirm their identities. Using genome walking, a larger (3,474 bp) AOX clone was isolated from vectorette libraries generated by digesting genomic DNA from MSC16 with one of five restriction enzymes (*Bam*HI, *Eco*RI, *Hin*dIII, *Hae*III, and *Eco*RV) followed by ligations with adapters compatible with these enzymes as described by the manufacturer (Sigma-Aldrich, St. Louis, MO, USA). The libraries were subjected to PCR using the vectorette-adapter-specific primers and cucumber AOX specific primers (OXF1 and OXR1, Table 1), according to the manufacturer’s protocol. The PCR reactions were diluted 1:100 and used as a template for an additional PCR using nested vectorette-specific and cucumber AOX-specific primers (OXF2 and OXR2, Table 1). Amplicons were cloned by TA tailing and Sanger sequenced (Genomed S.A., Warsaw, Poland). Further genome walking was completed using the vectorette libraries as described above and AOX-specific primers (Table 1).

**Table 1:** Oligonucleotide primer pairs used in this study to amplify and clone alternative oxidase (AOX) genes from cucumber and in RT-qPCR analysis

Primer	Sequence (5′ to 3′)	Description	Reference
P1	CTGTAGCAGCAGTVCCTGGVATGGT ^a^	Degenerated primers	Saisho et al. (1997)
P2	GGTTTACATCRCGRTGRTGWGCCTC ^a^		
OXF1	CTTCTTCAAGCAGGGCCTTAATCC	Vectorette primary primers	This study
OXR1	CTGCTCCCGCCATTGCCATCGACT		
OXF2	TGCTGAAACTTCCTCAGAGACTTC	Vectorette nested primers	
OXR2	GGATGCAAGGCTGAAGGATGTTAT		
AOX204	TCCACTGGAATCCACCATCCGACA	5′ RACE *AOX2* primers	
AOX92	CAGGCAAACTTAAAAGAACATCGAGCTA		
AOX389_39	ATGAATCGGATTGTGATTAGGAGTCTT	3′ RACE *AOX2* primers	
AOX822	TGGCTATTTGGAAGAGGAAGCGATCCA		
CsAox2FL_F	AATACGAGGGTAATTCACATCAACCAA	RT-qPCR primers Product size 126 bp	
CsAox2R101	TATGAAGCAAACCCCTGAGAAGAC		
CsAox2F1155	ATAGTACCTTGGTTTTGTGTTGTTGA	RT-qPCR primers Product size 169 bp	
CsAox2FL_R	CCAAGTAAACTTATGAAGCCCGACTTT		

^a^Codes for mixed base sites are R = A or G, W = A or T, and V = A or C or G

### 3′ and 5′ Rapid Amplification of cDNA Ends (RACE)

3′ and 5′ RACE was used to clone the full length of AOX2 cDNA from cucumber. Total RNA was isolated from MSC16 leaves and treated with DNaseI (Promega, Madison, WI, USA). 3′ and 5′ RACE was performed using GeneRacer RLM-RACE kit according to the manufacturer’s protocol (Invitrogen, Carlsbad, CA, USA). Primers specific for the alternative oxidase cDNA sequence were used for primary PCR—primer AOX389_39 for 3′ RACE and primer AOX204 for 5′ RACE, and for nested PCR primer AOX822 for 3′ end and AOX92 for 5′ RACE (Table 1). Full length AOX cDNA was PCR amplified from the total RNA to confirm the results. Sequence data from all clones were edited and contigs were aligned with Sequencer 5.1 (Gene Codes, Ann Arbor, MI, USA).

### Southern and Northern Blot Hybridization

Single 446 bp AOX clone was nick-translated and hybridized to DNA-gel blots of the cucumber line B, the melon (*Cucumis melo* L.) cultivar ‘Iroquois’, the watermelon (*Citrullus lanatus* [Thomb.] Mansf.) cultivar ‘Dixielee’, *Cucurbita pepo* L. ‘Dark Green Zucchini’, and *Cucurbita moschata* Duch. ‘Butternut’. In preliminary experiment DNAs of cucumber, melon, watermelon, and *Cucurbitas* were digested with three enzymes (*Eco*RI, *Hind*III, and *Xba*I). Additionally, cucumber line B DNA was digested with nine enzymes (*Bam*HI, *Bg*III, *Dra*I, *Eco*RI, *Eco*RV, *Hae*III, *Hind*III, *Sac*I, and *Xba*I). The nick-translated 446 bp AOX clone was also hybridized to RNA-gel blots prepared from total RNA isolated from leaves and flowers from line B (wild-type) and MSC16. Both hybridization analyses were performed as previously described by Bartoszewski et al. (2004).

### Cucumber Fosmid Library Screening

The cucumber fosmid library described by Meyer et al. (2008) was screened by filter hybridization. A single clone with 446 bp AOX fragment was hybridized with the library as described earlier. DNA of AOX-carrying fosmid was isolated by Qiagen Plasmid Maxi Kit (Qiagen, Hilden, Germany) and sequenced using 454 technology at the IBB PAN Sequencing and Oligo Synthesis Core (IBB Oligo, Warsaw, Poland).

### The Representative Set of AOX Proteins

A set of 42 sequences of AOX proteins was selected from NCBI GenBank and used in phylogenetic analyses, accession numbers: *Arabidopsis thaliana* AOX1a (NP_188876), AOX1b (NP_188875), AOX1c (NP_189399), and AOX2 (NP_201226); *Candida albicans* AOX0a (AAC98914) and AOX0b (AAF21993); *C. lanatus* AOX2 (AEN99850); *Cicer arietinum* AOX1 (XP_004512369), AOX2a (XP_004504796), and AOX2b (XP_004504799); *Cucumis sativus* AOX2 (AAP33163); *Chlamydomonas reinhardtii* AOX0a (AAG33633) and AOX0b (AAG33634); *Crassostrea gigas* AOX (ACL31211); *Glycine max* AOX1 (NP_001236166), AOX2a (AAB97285) and AOX2b (AAB97286); *Medicago sativa* AOX1 (AGQ42774), AOX2a (AGQ42775), AOX2b1 (AGQ42776), and AOX2b2 (AGQ42777); *Neurospora crassa* AOX (AAC37481); *Novosphingobium aromaticivorans* DSM 12444 AOX (YP_496850); *Trypanosoma brucei brucei* (XP_822944); *Vigna unguiculata* AOX1 (AAZ09196), AOX2a (ABM66368) and AOX2b (AAZ09195). The AOX2 protein from *C. melo* (MELO3C027020P1) was identified in a genomic database (http://melonomics.net/) and the AOX proteins from *Cajanus cajan*, *Lotus japonicus*, *Medicago truncatula*, and *Phaseolus vulgaris* were obtained from genomic databases as described by Cavalcanti et al. (2013). Classification of AOX proteins from higher plants, *C. albicans* and *C. reinhardtii*, was according to Considine et al. (2002). Classification of AOX proteins from the remaining organisms is consistent with the accession description at NCBI GeneBank records. Sequences of AOX proteins were used to construct the phylogenetic tree (Supplementary file 1).

### Genome-Wide Identification of Cucumber AOX Genes

To identify genomic regions carrying AOX, the representative set of 42 AOX protein sequences (described above) were blasted against local database with three whole-genome sequences of cucumber: line B10 (Wóycicki et al. 2011; http://csgenome.sggw.pl/), line 9930 (Huang et al. 2009; http://www.icugi.org/), and line Gy14 (Yang et al. 2012; http://cucumber.vcru.wisc.edu/) using default settings.

### Phylogenetic and Comparative Protein Analysis

A phylogenetic tree of the 42 AOX proteins from 12 different higher plants of the Cucurbitales, Fabales, and Brassicales; fungi (*N. crassa* and *C. albicans*); green algae (*C. reinhardtii*); eubacteria (*N. aromaticivorans*); protists (*T. brucei*), and animalia (*C. gigas*) was constructed by the neighbor-joining method (Saitou and Nei 1987) with bootstrap values (1,000 replicates) shown next to the branches (Felsenstein 1985) using the MEGA6 program (Tamura et al. 2013). The evolutionary distances were computed using the Poisson correction method (Zuckerkandl and Pauling 1965) and are in the units of the number of amino acid substitutions per site. All ambiguous positions were removed for each sequence pair.

Detailed analysis of the cucurbit AOX sequences (*Cucumis sativa*, *C. melo*, and *C. lanatus*) were performed using the model proposed by Costa et al. (2014). Due to the broad diversity in the first 100 amino acids of the AOX protein sequences for comparative analysis the conserved fragment of AOX sequence (101–354 aa) was used. For ‘sequence harmony’ (SH) methodology (Feenstra et al. 2007) the set of 67 AOX2a–c subtype proteins (prepared by Costa et al. (2014)) and SeqHarm version 1.1 (http://www.ibi.vu.nl/programs/seqharmwww/) was carried out. It is assumed that the SH score of 0.2 points out the sites which are rated as exhibiting relevant differences between the compared groups of sequences.

### Real-Time qPCR

Total RNA was isolated using Trizol method (Concert™ Plant RNA Reagent, Invitrogen). RNA was treated with DNaseI (TURBO DNA-free kit, Ambion Inc., Austin, TX, USA) and the PCR checked for lack of DNA contamination. The concentration and the quality of RNA (260/280 and 260/230 ratios) were measured before and after DNaseI treatment with the NanoDrop 2000 Spectrophotometer (Thermo Fisher Scientific, Waltham, MA, USA). The concentration of pure RNA was adjusted to 300 ng/μL. cDNA was synthesized using Transcriptor High Fidelity cDNA Synthesis Kit (Roche, Basel, Switzerland) in a 20-μl reaction using oligo(dT) primers according to manufacturer’s instructions.

Quantitative real-time reverse transcriptase PCR (RT-qPCR) method was applied to compare AOX transcript level in the MSC mutants and control line B (wild-type) plants grown in optimal phytotron conditions and in line B under cold stress. All RT-qPCR assays were performed using three biological replications with three technical replicates for each tested line and growth conditions. Analysis was carried out using diluted cDNA (1:11.5) reverse transcribed from 2.1 μg of total RNA. For RT-qPCR 4 μl of cDNA was used. The expression study was carried out using CFX96 Touch cycler (Bio-Rad Laboratories, Hercules, CA, USA) with Master Mix Maxima SybrGreen qPCR MM 2× ROX (Thermo Fisher Scientific, Waltham, MA, USA) according to the manufacturer’s instructions. RT-qPCR program was 50 °C for 2 min to activate Maxima DNA polymerase, 95 °C for 10 min, followed by 40 thermal cycles of 15 s at 95 °C and 1 min at 58 °C. Melting curve analysis was performed immediately after the RT-qPCR. The temperature range used for the melting curve generation was from 70 to 95 °C. Parameters of RT-qPCR were established by series of preliminary experiments with various PCR cycle numbers and annealing temperature gradients (55–65 °C).

Two pairs of primers designed to opposite ends of the AOX2 transcript were used: 5′ end pair complementary to 5′ UTR region of AOX2 gene (CsAox2FL_F and CsAox2R101) and 3′ end pair complementary to 3′ UTR region of AOX2 gene (CsAox2F1155 and CsAox2FL_R) (Table 1). The efficiency of amplification for all used RT-qPCR primers comprised within the range of 96.4–99.7 %.

Reference genes for data normalization were selected using the geNorm V3.4 applet (Vandesompele et al. 2002) from 13 candidate genes proposed for cucumber and tested in this study (Supplemental table S1). For stronger inference in both types of growth conditions, two reference genes were used. The most stable candidate reference genes for cucumber MSC mutants and control line B (wild-type) grown under phytotron conditions with the lowest M-value (*M* = 0.197) were *UBI*-*ep* (ubiquitin extension protein) and *TIP41* (TIP41-like protein family) and were used as reference genes (Supplemental table S2). For cold-treatment experiment, the greatest stability of expression (*M* = 0.486) was demonstrated for two genes, *M2* (cyclophilin) and *mdhG* (glyoxysomal malate dehydrogenase), primers for the experiment were provided by PrimerDesign Ltd (Southampton, UK) (Supplemental table S2).

The mean efficiency of amplification in RT-qPCR reactions was assessed using LinRegPCRv2013.1 (Ramakers et al. 2003) based on linear regression calculated for the slope of the regression line in the exponential growth phase of the product for each sample individually. Relative normalized expression (2^−ΔΔCt^ method) of *Aox2* transcript and statistical analysis (Student’s *t* test) to determine significance of differences were performed using the CFX Manager ™ software 3.1 (Bio-Rad Laboratories).

### Immunoblotting Analyses

#### For leaves and Flowers of Field Grown Plants

Total plant proteins were extracted from leaves and flowers collected from plants grown in the field. Briefly, collected tissue frozen in liquid nitrogen was ground using mortar and pestle and extracted in buffer (100 mM Tris, 200 mM KCl, 10 mM EDTA, 1 % (*v*/*v*) Triton X-100, and pH 8.0). Protein content was determined by the method of Bradford (Bradford 1976) and verified by SDS-PAGE and Coomassie staining. For immunoblotting analysis, 30–40 μg of total plant proteins were loaded on 15 % SDS PAGE gels, electrophoresed, and blotted on the Hybond C Extra nitrocellulose membrane (GE Healthcare, Little Chalfont, UK). Membranes were blocked using 5 % milk powder and probed with antibodies according to membrane manufacturer’s instructions. Both primary and secondary antibodies (anti-AOX 1:200 and anti-porin 1:500) were diluted in PBS containing 0.1 % Tween. Immunoreactive proteins were visualized by chemiluminescence on photographic film. Mouse monoclonal antibodies anti-AOX and anti-porin (Elthon et al. 1989; Blake et al. 2007) were kindly provided by Prof. Tom Elthon, University of Nebraska.

#### For Plants Grown Under Controlled Conditions

Total plant proteins were extracted in buffer (2 % *w*/*v* SDS, 50 mM TrisHCl, pH 7.6, and 1 mM β-mercaptoetanol). The final concentration of total protein was leveled with RC DC Protein Assay Kit (Bio-Rad Laboratories) according to manufacturer’s protocol. For immunoblotting analysis, 25 μg of total plant proteins were loaded on SDS PAGE gels (4 % stacking and 12 % running gel), electrophoresed, and transferred on the PVDF membrane (Bio-Rad Laboratories). Ponceau S (Sigma-Aldrich) membrane staining was performed as a loading control. Membranes were blocked using 5 % Blotting Grade Blocker Non Fat Dry Milk (Bio-Rad Laboratories), washed, and probed with antibodies: rabbit polyclonal antibodies anti-AOX1/2 (Agrisera, Vännäs, Sweden) and for cold experiment with line B also mouse monoclonal antibodies anti-Alpha-tubulin (Invitrogen). Antibodies were diluted anti-AOX1/2 1:1,000 and anti-Alpha-tubulin 1:250 in TBS-T and incubated with membrane for 1.5 h. Horseradish peroxidase conjugated goat anti-rabbit (1:25,000) or rabbit anti-mouse (1:5,000) antibodies (Agrisera) were used as secondary antibodies. Both were diluted in HST buffer (5 mM TrisHCl pH 7.5, 1 M NaCl, and 0.5 % (*w*/*v*) Tween 20) and incubated with membrane for 1 h. The chemiluminescence reaction was carried out using ECL Amersham Prime Western Blotting Detection Reagent (GE Healthcare) according to manufacturer’s protocol. Immunodetection was visualized directly on the membrane using ChemiDoc XRS+ system (Bio-Rad Laboratories). Western blots for cold-treated plants were performed in two biological replications with two technical replicates each. The results of experiment with cold-treated line B (wild-type) were quantified by densitometry (*n* = 4) using ChemiDoc XRS+ system (Bio-Rad Laboratories) and differences between successive time points after chilling were calculated by *t* Student test using Statistica version 10 (StatSoft, Tulsa, OK, USA).

## Results

### Cloning of AOX Genes from Cucumber

**Fig. 1:**
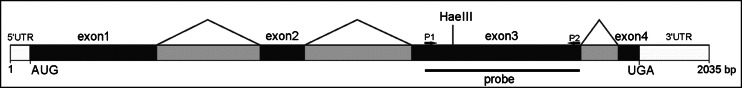
The structure of the cucumber AOX2 gene (NCBI Gen Bank Acc. No. AY258276). The cucumber AOX2 gene (2,035 bp) possesses four exons and three introns with 55 bp 5′ UTR and 200 bp 3′ UTR regions. P1 and P2 are degenerated primers used to amplify conserved 446 bp fragment of AOX gene (called ‘probe’, used in hybridization experiments)

Primers P1 and P2, designed to conserved regions of AOX proteins (Saisho et al. 1997), amplified a single 446 bp fragment from cucumber. DNA of 15 putative AOX clones from two independent PCR reactions were sequenced to confirm their identities. All sequenced clones were identical, suggesting that cucumber possesses a single AOX gene. A larger (3,474 bp) genomic clone of AOX was isolated from vectorette libraries of cucumber. 3′and 5′ RACE was used to clone the full length AOX transcript. Based on the genomic (2,035 bp) and full length cDNA (1,551 bp) sequences, the structure of cucumber AOX gene was established (Fig. 1). The sequence of AOX gene with coding sequence was deposited in NCBI as accession AY258276. The cucumber AOX gene possesses four exons and three introns with 55 bp 5′ UTR and 200 bp 3′ UTR regions, similar to other plants (Fig. 1).

### Cucumber Possesses a Single AOX Gene

**Fig. 2:**
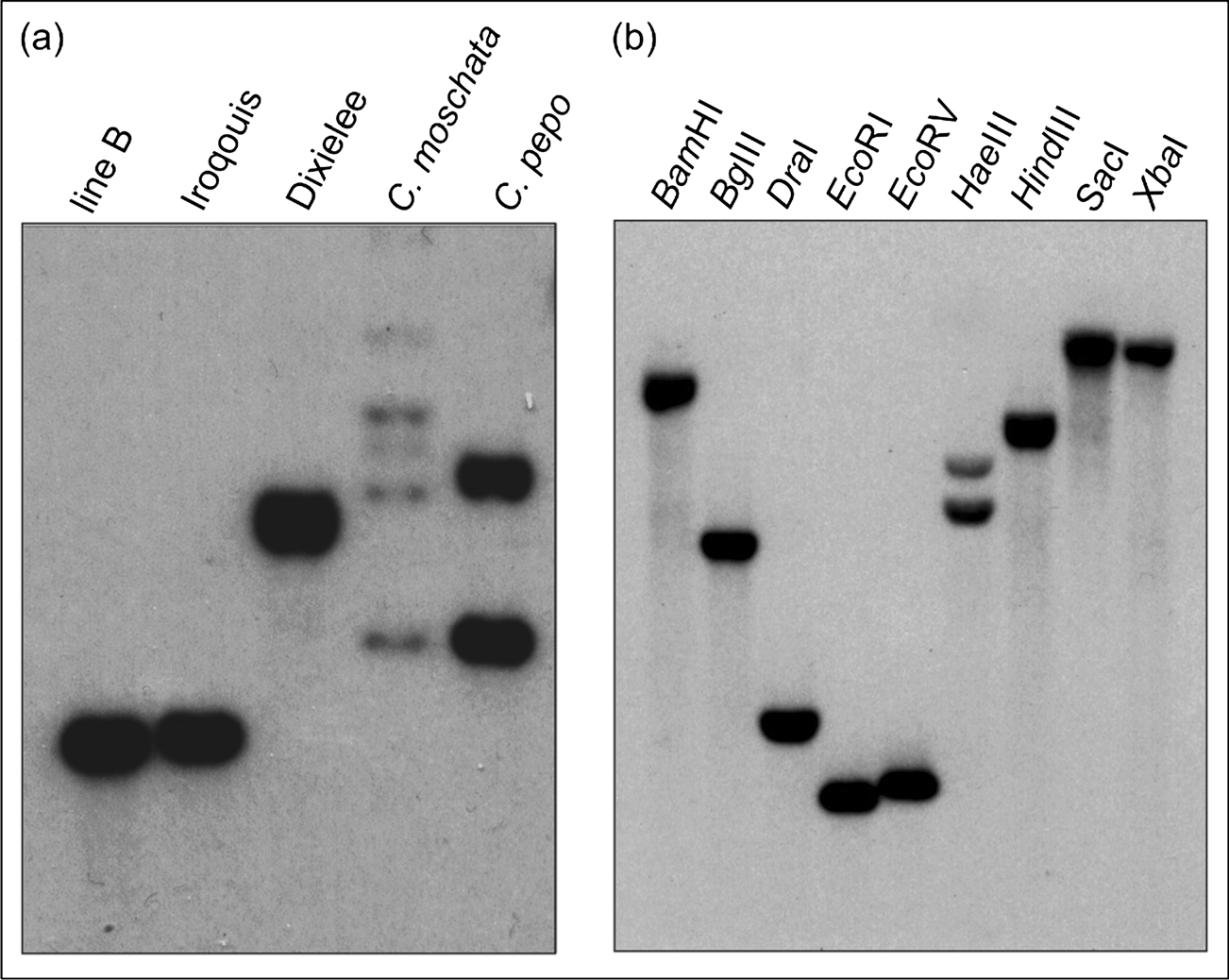
Southern blot hybridizations from hybridization of nick-translated 446 bp AOX clone (‘probe’, Fig. 1) as an evidence for the presence of a single AOX gene in cucumber. **a** Cucumber line B, melon ‘Iroqouis’, watermelon ‘Dixielee’, *Cucurbita moschata* ‘Butternut’, and *Cucurbita pepo* ‘Dark Green Zucchini’ DNA digested with *Eco*RI and **b** cucumber line B DNA digested with nine different restriction enzymes

Southern hybridization of exon 3 of AOX (Saisho et al. 1997, Fig. 1) was used to assess numbers of AOX genes in the cucurbits. The results revealed a single band for cucumber, melon, and watermelon. However, three major and two weaker bands were detected for *C. moschata* and two bands for *C. pepo* (Fig. 2a). When the same probe was hybridized to cucumber, DNAs singly digested with nine enzymes revealed a single band for eight restriction enzymes and two bands for *Hae*III, revealing an internal *Hae*III restriction site within the probe region (Fig. 2b). Presence of this *Hae*III site was confirmed later by *in silico* restriction sites analysis in the genomic sequence. The same AOX exon three probe was hybridized to the cucumber fosmid library and the single clone carrying the AOX gene was identified. This AOX-carrying fosmid was sequenced and contained the same AOX sequence as cloned by PCR. These results strongly support a single AOX gene in cucumber, melon, and watermelon, and more than one AOX gene in *Cucurbita* (the representative results for *Eco*RI digestion are shown in Fig. 2a).

### Cucumber, Melon, and Watermelon AOX Genes Encode AOX2

**Fig. 3:**
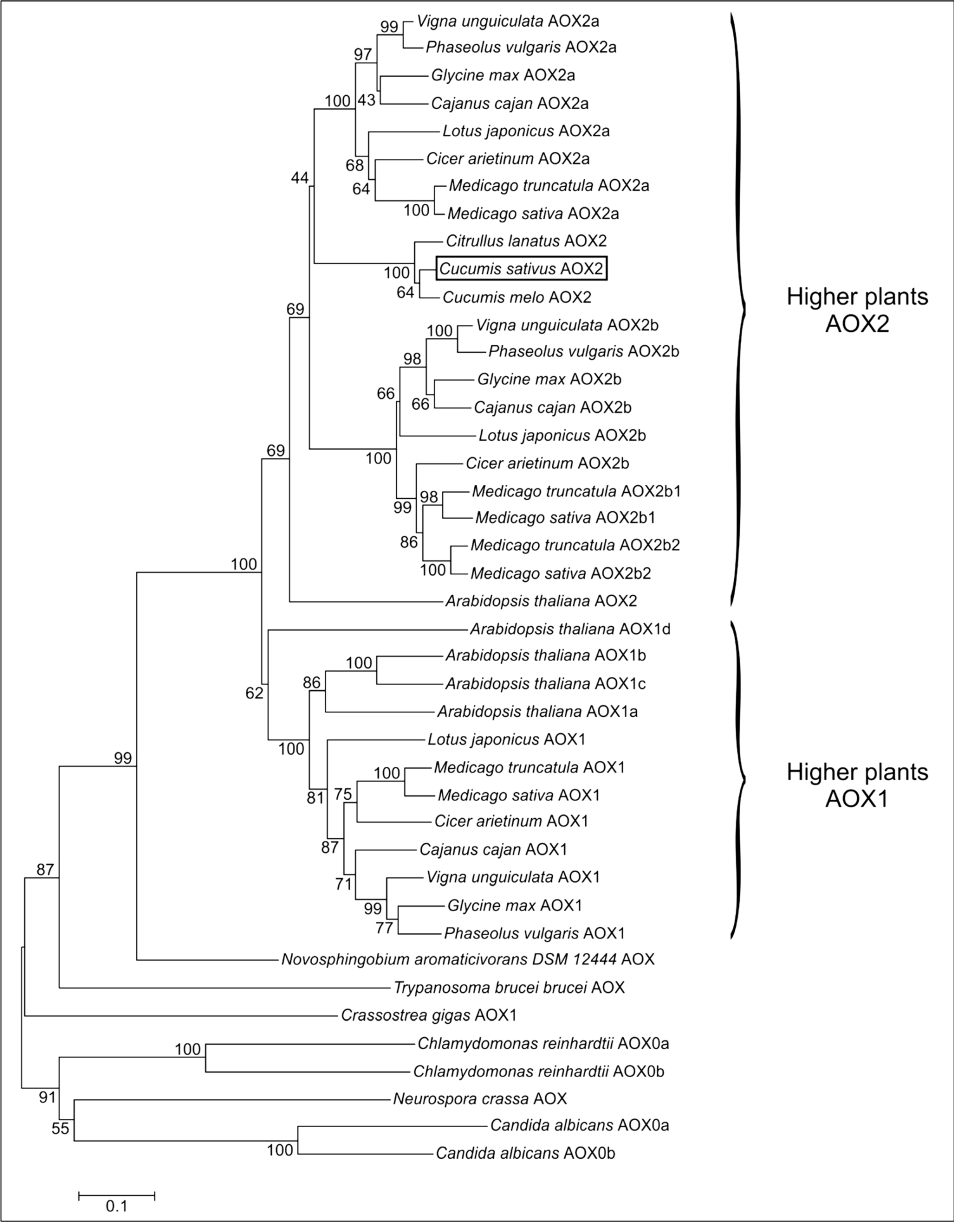
Phylogenetic tree of 42 AOX proteins from 12 different higher plant species of the *Cucurbitales*, *Fabales*, and *Brassicales*; fungi (*N. crassa* and *C. albicans*); green algae (*C. reinhardtii*); eubacteria (*N. aromaticivorans*); protists (*T. brucei*), and animalia (*C. gigas*). Classification of higher plants, *C. albicans*, and *C. reinhardtii* AOX proteins is according to Considine et al. (2002). Classification of AOX proteins from other organisms is consistent with the protein sequence description at the NCBI GeneBank. The tree was obtained by the Neighbor-Joining method with 1,000 bootstrap replicates using the MEGA6 program (Tamura et al. 2013). *Branches* are drawn in proportion to genetic distance according to the scale shown in the *bottom of the figure*. The tree was constructed according to sequence data indicated in the Supplementary file 1

Based on the full length cDNA sequence, the protein sequence of cucumber AOX was predicted to have 346 amino acids (NCBI GeneBank Accession APP33163). The optimal phylogenetic tree based on the amino acid sequences of 42 AOX proteins from diverse monocots and dicots had sum of branch length 6.686 (Fig. 3). Cucumber AOX formed a new branch and was placed with AOX proteins from melon and watermelon in the higher plants AOX2 cluster, between AOX2a and AOX2b subgroup as distinguished by Considine et al. (2002) (Fig. 3).

### Identification of Amino Acids Specific to AOX2 in Cucumber, Melon, and Watermelon

**Fig. 4:**
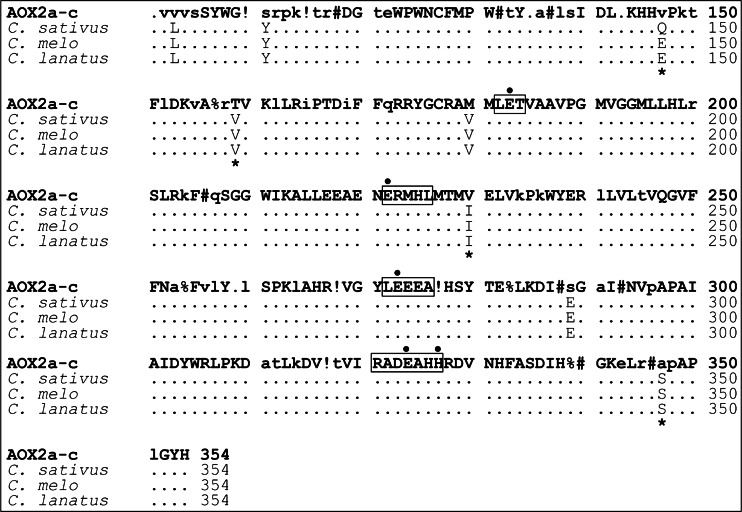
Amino acid sites unique to AOX2 in cucumber, melon, and watermelon. An alignment of the conservative fragment (101–354 aa) of AOX2a–c consensus sequence (Costa et al. 2014) and cucumber, melon, and watermelon corresponding AOX fragments appear with eight specific amino acids residues. Indicated amino acids sites were rated as exhibiting relevant differences using the ‘sequence harmony’ (SH) methodology (Feenstra et al. 2007). In addition, those presenting the strong reliability parameters in SH ≤ 0.13 are marked with an *asterisk*. Figure was prepared by using the model proposed by Costa et al. (2014). Four highly conserved AOX active regions (LET, NERMHL, LEEEA, and RADE__H) were *marked by frames*, glutamic acid (E) and histidine (H) amino acid residues involved in iron-binding were indicated by *black circles* (Berthold et al. 2000). *Capital letters at the specific sites* represent the most frequently seen amino acids. Consensus symbols used in the AOX2a–c consensus sequences: *exclamation mark* is I (Isoleucine), or V (valine); *percent sign* is F (phenylalanine), or Y (tyrosine); *number sign* is N (asparagine), D (aspartic acid), Q (glutamine), or E (glutamic acid). For more details, the reader is referred to the Supplementary file 2

Analysis of the protein sequences based on the classification proposed by Costa et. al. (2014) showed that the AOX2 proteins of *C. sativus*, *C. melo*, and *C. lanatus* have almost all the amino acids specific for the AOX2a–c subtype, but are also polymorphic for 16 amino acids sites within the conservative AOX region. The specificities of eight sites were confirmed using ‘sequence harmony’ (SH) methodology (Feenstra et al. 2007). Four of the differences possess relatively strong reliability parameters in SH ≤ 0.13 and four relevant differences SH = 0.17 (AOX2a–c consensus positions: 147, 159, 230, 347 and 103, 111, 180, 289, respectively) (Fig. 4, Supplemental table S3, and Supplementary file 2).

Although none of the eight polymorphic amino acids are located in the four highly conserved regions of AOX, some are close to highly conserved and functionally important amino acids. Amino acids in position 103, 111, and 147* (* indicates positions with relatively strong reliability parameters at SH ≤ 0.13) are in the neighborhood of conserved cysteine residue CysI (C127) associated with post-translational modifications (Berthold et al. 2000; Costa et al. 2014). Residues in position 159*, 289, and 347* are located close to the second conserved cysteine CysII (C177), LEEEA region, and RADE__H region, respectively. Two amino acids situated in position 180 and 230* were found in close proximity to the functional regions: residue at position 180 is situated between second highly conserved cysteine CysII (C177) and the LET region (involved in iron-binding) and amino acid at position 230* is situated close to NERMHL region (utilized to form the four-helix bundle required for iron-binding) (Berthold et al. 2000). Therefore, cucumber, melon, and watermelon possess a single AOX belonging to the AOX2a–c group with several unique amino acid residues.

### Chromosomal Localization of Cucumber AOX2

Using the three available cucumber genomic sequences [line 9930 (Huang et al. 2009), line Gy14 (Yang et al. 2012), and line B10 (Wóycicki et al. 2011)], we confirmed that there is only one AOX2 gene in the cucumber genome and no AOX1 genes. Analysis of the genomes with chromosomal versions available (line 9930 and Gy14) showed that AOX2 gene is located in the middle of the upper arm of chromosome 4 (Table 2).

**Table 2:** Chromosomal positions of AOX2 gene in the cucumber genome line 9933 version 2 (http://www.icugi.org/) and Gy14 assembly version 1 (http://cucumber.vcru.wisc.edu/) based on BLAST search

Chromosome	AOX2 gene region	Line 9930 genome position (Csa4M109010.1)		Line Gy14 genome position (Cucsa.398150.1)		Starting gene position	Ending gene position
		Starting	Ending	Starting	Ending		
Chr 4^a^	5′ UTR	6,991,492	6,991,438	6,688,767	6,688,713	−55	−1
	Exon1	6,991,437	6,991,075	6,688,712	6,688,350	1	363
	Intron1	6,991,074	6,990,769	6,688,349	6,688,044	364	669
	Exon 2	6,990,768	6,990,640	6,688,043	6,687,915	670	798
	Intron 2	6,990,639	6,990,322	6,687,914	6,687,597	799	1,117
	Exon 3	6,990,321	6,989,833	6,687,596	6,687,108	1,118	1,607
	Intron 3	6,989,832	6,989,718	6,687,107	6,686,993	1,608	1,723
	Exon 4	6,989,717	6,989,658	6,686,992	6,686,933	1,724	1,784
	3′ UTR	6,989,657	6,989,458	6,686,932	6,686,733	1,785	1,985
	AOX fosmid	7,024,359	6,983,645	6,720,115	6,681,175	1	40,869

^a^Estimated size of chromosome 4 of line 9930 is 23,425,844 and Gy14 is 23,376,920 bp, AOX2 gene is present in the reverse orientation in both genomes

### Expression of AOX2

**Fig. 5:**
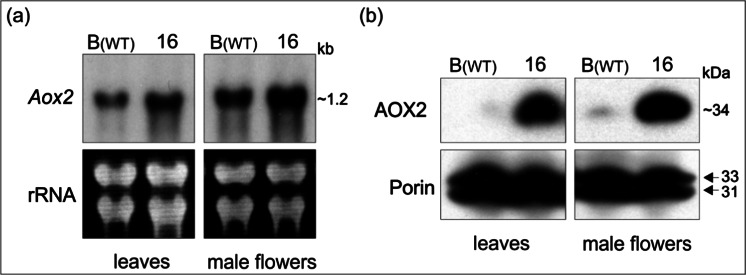
Expression of AOX2 in field grown plants of cucumber and in leaves and flowers of control line B (wild-type) and mutant MSC16. Revealing higher amounts of *Aox2* transcripts by Northern-blot analysis **a** and AOX2 protein by Western blot **b** in MSC16. **a** Relative amounts of alternative oxidase transcript in total RNA extracts. Approximate sizes of *Aox2* transcript in kilobases is shown on *right. Gel picture at bottom* shows ethidium bromide stained rRNA showing equal loading of RNA samples. **b** Relative amounts of alternative oxidase and porin in total protein extracts. Approximate sizes of polypeptides in kilodaltons are shown on *right*. Porin was used as internal control

Initial comparative analysis performed for field-grown plants of line B (wild-type) and mitochondrial mutant MSC16 revealed that cucumber *Aox2* transcripts were detected both in leaves and in male flowers with a higher level in MSC16 (Fig. 5a). Western blots confirmed this result showing higher AOX2 abundance in the mitochondrial mutant MSC16 (Fig. 5b).

**Fig. 6:**
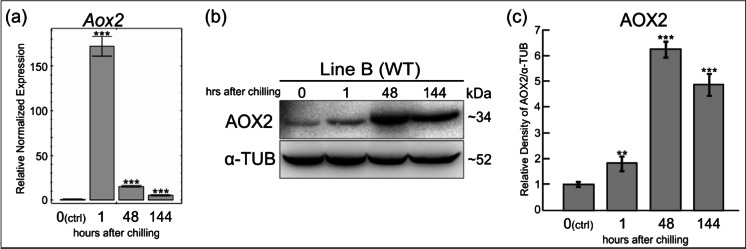
Expression AOX2 in cucumber line B after cold treatment. Both analyses RT-qPCR (**a**) and Western blot (**b** and **c**) revealed that cucumber AOX2 is cold-responsive. Rapid increase (172-fold, *p* < 0.001) of *Aox2* transcript abundance after chilling was observed (**a**) and the significantly highest amount of AOX2 protein (6.3-fold increase, *p* < 0.001) was proved 48 h (2 days) after chilling (**b** and **c**). Differences between stages after chilling were referenced to the state before chilling marked as ‘0(ctrl)’. The following ranges of *P* value were defined: *p < 0.05, **p < 0.01, and ***p < 0.001. **a** RT-qPCR results obtained with CsAox2FL_F and CsAox2R101 primers designed to 5′ end of *Aox2*. Primers specific to *UBI*-*ep* and *TIP41* were used as reference genes (Supplemental tables S1 and S2). The diagram shows the average relative normalized expression to control (pre-chilling). *Bars* represent standard error of the mean (SEM). **b** Western blot confirmed cold regulation of AOX2. Alpha-tubulin was used as an internal control. Approximate size of polypeptides in kilodaltons are shown on *right*. **c** Densitometry quantification of Western blot analysis (*n* = 4) performed using ChemiDoc XRS+ system (Bio-Rad Laboratories). Relative amount of AOX2 was normalized to alpha-tubulin

Detailed analysis of the expression of AOX2 in cucumber line B (wild-type) at the transcript and protein level showed that the *Aox2* gene is upregulated by cold stress (Fig. 6). The amount of *Aox2* transcripts was increasing rapidly (172-fold, *p* < 0.001) immediately after chilling and decreasing with each passing day from the occurrence of cold stress (Fig. 6a). Western blot confirmed that cucumber AOX2 is upregulated by cold stress and statistically significant differences were revealed (Fig. 6b, c). The amount of AOX2 significantly increased 1 h after chilling (1.8-fold, *p* < 0.05) compared to the AOX2 amount before cold treatment. The highest level of AOX2 was observed 48 h (2 days) after chilling (6.3-fold increase, *p* < 0.001). With time, the amount thereof was reduced, wherein the level 144 h (6 days) after cold treatment was still relatively high compared to the level before chilling (4.9-fold increase, *p* < 0.001) (Fig. 6b, c).

**Fig. 7:**
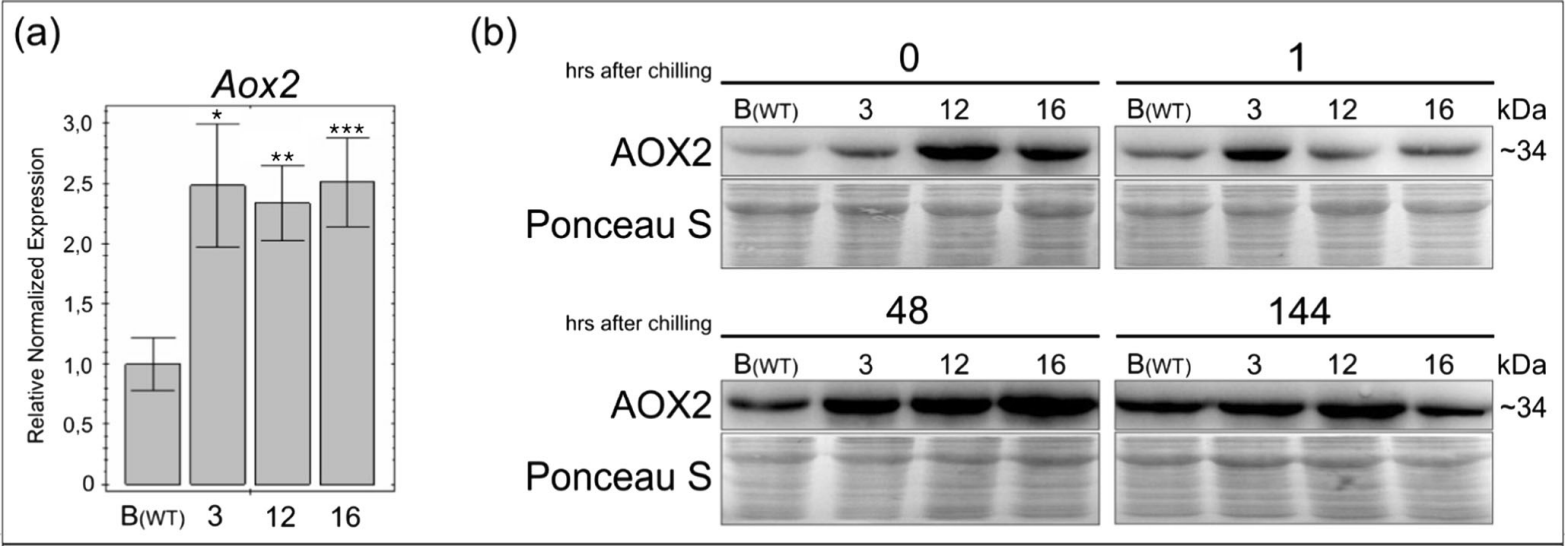
Expression of AOX2 in cucumber control line B (wild-type) and MSC lines. AOX2 is upregulated in MSC lines 3, 12, and 16 both before and after chilling compared to control line B (B_WT_). **a** Results of RT-qPCR analysis performed with primers designed to 5′ end of *Aox2* (CsAox2FL_F and CsAox2R101). Primers specific to *M2* and *mdhG* were used as reference genes (Supplemental table S1 and S2). The diagram shows *Aox2*, the average relative expression in MSC mutants before chilling, referenced to the control line B (B_WT_, referred to as 1). *Bars* represent standard error of the mean (SEM). Statistical differences were confirmed using *t* Student test and *P* value < 0.05 was considered as statistically significant. The following *P* value ranges were defined: *p < 0.05, **p < 0.01, and ***p < 0.001. **b** AOX2 expression pattern in the MSC mutants under cold stress. Ponceau S staining was used as a loading control. Approximate size of AOX2 in kilodaltons is shown on *right*

Extended comparative analysis performed for both the line B (wild-type) and MSC lines 3, 12, and 16 showed that all MSC lines grown in optimal conditions revealed elevated expression level of *Aox2* transcripts (2.3- to 2.5-fold increase, at least *p* < 0.05) relative to untreated wild-type, control line B (Fig. 7a). No significant differences were observed in *Aox2* expression among the MSC lines (Fig. 7a). Moreover, Western blot analysis demonstrated that in all MSC lines 3, 12, and 16 AOX2 protein was detected both before and after cold treatment at a consistently high level with the highest amount observed 48 h (2 days) after cold treatment (Fig. 7b), which is similar to observations made for control line B.

## Discussion

Two AOX subfamilies (AOX1 and AOX2) are present in angiosperms (Considine et al. 2002). In monocots, duplications of AOX1 gene are common and AOX2 subfamily was lost early in evolution (Costa et al. 2014). In eudicots, both subfamilies AOX1 and AOX2 are present. Different AOX1 subtypes being a result of duplications are well known and duplications of AOX2 were recently classified into two subtypes—AOX2a–c and stress-responsive AOX2d (Costa et al. 2014).

In this study, we identified a single AOX2 gene after carefully evaluating three available genomic sequences of cucumber (line 9930, line Gy14, and line B10), performing Southern blot hybridizations, and screening a cucumber fosmid library. There was no evidence for AOX1 gene(s) and no evidence for AOX2 gene duplication(s) in cucumber. The alignment of the consensus sequences of AOX2a–c (Costa et al. 2014) and cucumber, melon, and watermelon AOX proteins demonstrates agreement for all residues specific for AOX2a–c subtype. However, we identified variation for 16 specific amino acids in conserved regions of the AOX2 protein (101–354 aa). Eight were identified as exhibiting relevant differences between the consensus sequence of AOX2a–c subtype (Costa et al. 2014) and cucumber, melon, and watermelon using the ‘sequence harmony’ (SH) methodology (Feenstra et al. 2007). Although six of the differences were not located in the immediate vicinity of the functional regions (Berthold et al. 2000; Costa et al. 2014), their effect on AOX2 protein structure could not be excluded. Two amino acids (position: 180, 230*) were located close to important functional regions of AOX at position 180 situated between the second highly conserved cysteine CysII (C177) and the LET region (involved in iron-binding) and position 230* close to NERMHL region utilized to form the four-helix bundle required for iron-binding (Berthold et al. 2000). Both substitutions demonstrated compliance with residues specific for AOX1d: 180 with residue found in monocot AOX1d and 230* residue frequently occurring in eudicot AOX1d (Costa et al. 2014). These findings indicate that cucumber likely lost AOX1 gene(s) and did not experience AOX2 duplication(s), resulting in a single AOX2 gene that assumes the functions of AOX1 gene(s) for stress responsiveness. However, it is also possible that cucumber AOX2 gene is the primary AOX gene that did not undergo any divergence and its expression covers all AOX functions.

Besides the AOX1 genes, there are examples of AOX2 genes regulated by stress in *A. thaliana* and leguminous species (Clifton et al. 2005; Costa et al. 2010; Cavalcanti et al. 2013). However, all stress-regulated AOX2 genes were classified based on protein similarity into AOX2d subtype (Costa et al. 2014). In this study, we provide evidence that the cucumber AOX2 of AOX2a–c subtype is regulated by cold stress, induced by chilling in control line B (wild-type) and upregulated before and after cold treatment in the three MSC mitochondrial mutants. The study of Lei et al. (2010) revealed that cucumber seedlings treated with combination of low temperature and SA show enhanced expression and accumulation of AOX but did not define what AOX. In this study we proved that cucumber has only one AOX2a–c. This clearly shows that AOX2a–c genes can be stress-responsive. This finding extends the observation that only AOX2d genes can be stress-responsive (Costa et al. 2014). The findings that AOX2 in cucumber is stress-responsive (chilling), SA regulated, belongs to AOX2a–c subtype with specific amino acids residues demonstrate that the cucumber AOX2 functions uniquely from previously described AOX2 genes.

Juszczuk et al. (2007) and Szal et al. (2009) studied hydroponically grown cucumber and showed that the AOX protein level was higher in MSC16 compared to wild-type line B. Florez-Sarasa et al. (2009) demonstrated that despite higher AOX abundance, there was no difference in AOX activity in wild-type line B and MSC16 mutant. When these results were published, it was not known that cucumber possesses only a single AOX gene and therefore it was not possible to recognize that observed differences are effects of expression of the AOX2 gene and not AOX1. Moreover, the study was performed on a single mitochondrial mutant (MSC16); in our study we showed that three distinct MSC mutants characterized by unique rearrangements of their mtDNA (Bartoszewski et al. 2004) express higher level of AOX2. We also demonstrated that elevated levels of AOX2 protein is characteristic for all three MSC lines both before and after chilling.

We demonstrated that previously described classifications for alternative oxidases in higher plants do not fit cucumber, because of (1) the presence of a single AOX gene assigned to the subtype AOX2a–c, (2) AOX2 expression in cucumber is induced by cold stress suggesting it carries stress-related functions. We also found that three distinct MSC mutants, including MSC16 that possesses negatively affected respiratory complex I and increased ROS level (Juszczuk et al. 2007; Szal et al. 2009), show upregulation of AOX2a–c. These findings revealed that cucumber represents an interesting model to study the regulation of AOX expression, especially in the acclimation of plants to different environmental conditions. To verify whether the single cucumber AOX2a–c subtype is stress-responsive we decided to use cold treatment because cucumber is a thermophilic plant sensitive to low temperatures. It must be noted that elevated AOX expression was observed in a broad range of stress conditions (Clifton et al. 2005) and therefore, the next step should be taken to verify the regulation of the cucumber AOX2 expression by other stresses. However, it should be emphasized that this is the first report showing in a simple and clear way that the alternative oxidase from AOX2a–c subtype may respond to chilling stress. Also identification of cucumber AOX2 mutants or development of transgenic lines with downregulated and overexpressed AOX2 would benefit further studies. Recent studies indicate that the regulation of the AOX1a in *A. thaliana* not only alters auxin homeostasis, allowing plants to respond rapidly to environmental changes but also regulates the growth and development (Ivanova et al. 2014). Therefore, it is possible that increased expression of AOX2 in the cucumber MSC mitochondrial mutants can alter auxin homeostasis and result in their slower germination, weaker growth, and organ deformation.

There are several studies providing insights about nuclear response to mitochondrial stress where mitochondrial mutants of model plant species, including *Arabidopsis*, tobacco, and maize, were used (Newton et al. 1990; Kanazawa et al. 1994; Kuzmin et al. 2004; Shedge et al. 2007, 2010). The disadvantage of these studies is that these plant species possess maternally transmitted both chloroplast and mitochondria that make it difficult to distinguish clearly chloroplast from mitochondrial effects. Cucumber possesses paternally transmitted mitochondria, making it possible to separate putative chloroplast and mitochondrial effects at the genetics level by reciprocal crosses. Another limitation in mitochondrial retrograde signaling studies is that very few mitochondrial mutants are available and there is no effective method to obtain mitochondrial mutants. In cucumber, passage of wild-type cucumber through tissue cultures may be applied to develop lines possessing distinct mtDNA rearrangements (Malepszy et al. 1996) and it has been postulated as a method to generate mitochondrial mutants (Bartoszewski et al. 2007). Such mitochondrial mutants could be interesting plant material in the study of mitochondria to nucleus cross-talk. MSC lines used in this study were developed this way. Thus, we think that cucumber is an interesting plant species to study retrograde signaling.

In conclusion, our study showed that cucumber possesses a single AOX2 gene that is stress-regulated making it easy to manipulate and interpret AOX expression. Availability of the MSC mitochondrial mutants of cucumber with upregulated expression of AOX2 even in optimal growth conditions makes cucumber an intriguing model to study AOX expression and mitochondrial retrograde signaling. A full understanding of the mechanisms related to mitochondrial retrograde signaling in crop species could lead to discovery of novel stress–response genes as potential targets for plant breeding.

## Electronic supplementary material

Supplementary file 1 A text file containing a set of 42 AOX protein sequences in FASTA format. (DOCX 21 kb)

Supplementary file 2 Amino acids sites specific to AOX2 in cucumber, melon, and watermelon. An alignment of the conservative fragment (100–354 aa) of AOX2a–c consensus sequence (Costa et al. 2014) and cucumber, melon, and watermelon corresponding AOX fragments. (DOCX 41 kb)

Supplemental table S1 Description and primer sequences of candidate genes tested as potential RT-qPCR references for cucumber line B (wild-type) and MSC mutants grown under optimal and cold stress growth conditions. (DOCX 19 kb)

Supplemental table S2 Average gene expression stability values (M) of 13 tested RT-qPCR reference candidate genes in the study of *AOX2* expression in (a) cucumber MSC mutants grown in the optimal phytotron conditions and (b) cold-treated line B. (DOCX 18 kb)

Supplemental table S3 Specific amino acids of AOX2a–c and cucurbits AOX protein (*C. sativus*, *C. melo*, and *C. lanatus*) groups detected through the sequence harmony program (SeqHarm version 1.1) using a cutoff 0.2. (DOCX 29 kb)
