# Supplementary material for: Cucumber Possesses a Single Terminal Alternative Oxidase Gene That is Upregulated by Cold Stress and in the Mosaic (MSC) Mitochondrial Mutants
Source: Plant Mol Biol Report. 2015 Apr 21;33:1893–906. doi: 10.1007/s11105-015-0883-9 (PMC4695503; doi:10.1007/s11105-015-0883-9)
Supplement: Supplementary file 1 — A text file containing a set of 42 AOX protein sequences in FASTA format. (DOCX 21 kb) [file 11105_2015_883_MOESM1_ESM.docx]

**Cucumber possesses a single terminal alternative oxidase gene that is upregulated by cold stress and in the mosaic (MSC) mitochondrial mutants**

Journal: Plant Molecular Biology Reporter

Authors: Tomasz L. Mróz^A^, Michael J. Havey^B^, Grzegorz Bartoszewski^*A^

^A^Department of Plant Genetics, Breeding and Biotechnology, Faculty of Horticulture, Biotechnology and Landscape Architecture, Warsaw University of Life Sciences, ul. Nowoursynowska 159, 02-776 Warsaw, Poland

^B^Agricultural Research Service, U.S. Department of Agriculture, Vegetable Crops Unit, Department of Horticulture, 1575 Linden Dr., University of Wisconsin, Madison, WI 53706, USA

*email: grzegorz_bartoszewski@sggw.pl

**Supplementary file 1** The set of 42 AOX protein sequences in FASTA format.

>Arabidopsis_thaliana_AOX1a

MMITRGGAKAAKSLLVAAGPRLFSTVRTVSSHEALSASHILKPGVTSAWIWTRAPTIGGMRFASTITLGEKTPMKEEDANQKKTENESTGGDAAGGNNKGDKGIASYWGVEPNKITKEDGSEWKWNCFRPWETYKADITIDLKKHHVPTTFLDRIAYWTVKSLRWPTDLFFQRRYGCRAMMLETVAAVPGMVGGMLLHCKSLRRFEQSGGWIKALLEEAENERMHLMTFMEVAKPKWYERALVITVQGVFFNAYFLGYLISPKFAHRMVGYLEEEAIHSYTEFLKELDKGNIENVPAPAIAIDYWRLPADATLRDVVMVVRADEAHHRDVNHFASDIHYQGRELKEAPAPIGYH

>Arabidopsis_thaliana_AOX1b

MMMSRRYGAKLMETAVTHSHLLNPRVPLVTENIRVPAMGVVRVFSKMTFEKKKTTEEKGSSGGKADQGNKGEQLIVSYWGVKPMKITKEDGTEWKWSCFRPWETYKSDLTIDLKKHHVPSTLPDKLAYWTVKSLRWPTDLFFQRRYGCRAMMLETVAAVPGMVGGMLVHCKSLRRFEQSGGWIKALLEEAENERMHLMTFMEVAKPNWYERALVIAVQGIFFNAYFLGYLISPKFAHRMVGYLEEEAIHSYTEFLKELDNGNIENVPAPAIAIDYWRLEADATLRDVVMVVRADEAHHRDVNHYASDIHYQGRELKEAPAPIGYH

>Arabidopsis_thaliana_AOX1c

MITTLLRRSLLDASKQATSINGILFHQLAPAKYFRVPAVGGLRDFSKMTFEKKKTSEEEEGSGDGVKVNDQGNKGEQLIVSYWGVKPMKITKEDGTEWKWSCFRPWETYKADLTIDLKKHHVPSTLPDKIAYWMVKSLRWPTDLFFQRRYGCRAIMLETVAAVPGMVGGMLMHFKSLRRFEQSGGWIKALLEEAENERMHLMTFMEVAKPKWYERALVISVQGVFFNAYLIGYIISPKFAHRMVGYLEEEAIHSYTEFLKELDNGNIENVPAPAIAVDYWRLEADATLRDVVMVVRADEAHHRDVNHYASDIHYQGHELKEAPAPIGYH

>Arabidopsis_thaliana_AOX2

MSQLITKAALRVLLVCGRGNCNMFVSSVSSTSVMKSPYEITAPMRIHDWCGGFGDFKIGSKHVQGNFNLRWMGMSSASAMEKKDENLTVKKGQNGGGSVAVPSYWGIETAKMKITRKDGSDWPWNCFMPWETYQANLSIDLKKHHVPKNIADKVAYRIVKLLRIPTDIFFQRRYGCRAMMLETVAAVPGMVGGMLLHLKSIRKFEHSGGWIKALLEEAENERMHLMTMMELVKPKWYERLLVMLVQGIFFNSFFVCYVISPRLAHRVVGYLEEEAIHSYTEFLKDIDNGKIENVAAPAIAIDYWRLPKDATLKDVVTVIRADEAHHRDVNHFASDIRNQGKELREAAAPIGYH

>Arabidopsis_thaliana_AOX1d

MSYRSIYRTLRPVLSSSVQSSGLGIGGFRGHLISHLPNVRLLSSDTSSPVSGNNQPENPIRTADGKVISTYWGIPPTKITKPDGSAWKWNCFQPWDSYKPDVSIDVTKHHKPSNFTDKFAYWTVQTLKIPVQLFFQRKHMCHAMLLETVAAVPGMVGWMLLHLKSLRRFEHSGGWIKALLEEAENERMHLMTFIELSQPKWYERAIVFTVQGVFFNAYFLAYVISPKLAHRITGYLEEEAVNSYTEFLKDIDAGKFENSPAPAIAIDYWRLPKDATLRDVVYVIRADEAHHRDINHYASDIQFKGHELKEAPAPIGYH

>Cajanus_cajan_AOX1

MMMMMLSRGGSNRALLAAKGLSAEVGALKKWSYGVRSESTLAFSEKEKKVDSSSSAAAAADGKKEEKGIASYWGIEASKITKPDGTEWKWNCFRPWETYKADVSIDLKKHHAPVTFLDKMAFWTVKALRYPTDVFFQRRYGCRAMMLETVAAVPGMVGGMLLHCKSLRRFEHSGGWIKALLEEAENERMHLMTFMEVAKPKWYERALVITVQGVFFNAYFLGYLLSPKFAHRMVGYLEEEAIHSYTEFLKELDKGNIENVPAPAIAIDYWQLPPDSTLRDVVMVVRADEAHHRDVNHFASDIHYQGRELRDAAAPIGYH

>Cajanus_cajan_AOX2a

MKLLALSSAARRALLNGRNCNRHGSAAVRPFAAVETRLLCAGGGNGRFFYWKRTMASPAEAKMPEKEKDKEETEKSVAESSYWGISRPRVVREDGTEWPWNCFMPWETYRPNLSIDLTKHHVPKNFQDKVAYRTVKLLRIPTDVFFKRRYGCRAMMLETVAAVPGMVGGMLLHLKSLRKFENSGGWIKALLEEAENERMHLMTMVELVKPTWYERLLVLAVQGVFFNAFFVLYILSPKVAHRIVGYLEEEAIHSYTEYLKDLESGAIENVPAPAIAIDYWRLPKDAKLKDVITVIRADEAHHRDVNHFASDIHFQGKELREAPAPIGYH

>Cajanus_cajan_AOX2b

MKHILVRSAARVLLGGGRSYYRQLSTAPIVETRHQHGGAAFGSFYLRRMSSLPEIKDHQQSEEKKNEVNDANHNNAVISSYWGITRPKVRREDGTEWPWNCFMPWDSYRADVSIDVTKHHTPKSLTDKVAFRSVKFLRVLSDLYFKERYGCHAMMLETIAAVPGMVGGMLLHLKSLRKFQHSGGWIKALLEEAENERMHLMTMVELVKPSWHERLLIFTAQGVFFNAFFVFYLLSPKAAHRFVGYLEEEAVISYTQHLDAIESGKVENVPAPAIAIDYWRLPKDATLKDVVTVIRADEAHHRDVNHFASDIHHQGKELKEAPAPIGYH

>Candida_albicans_AOX0a

MIGLSTYRNLPTLLTTTTVISTALRSKQLLRFTTTTSTKSRSSTSTAATTVGNSNPKSPIDEDNLEKPGTIPTKHKPFNIQTEVYNKAGIEANDDDKFLTKPTYRHEDFTEAGVYRVHVTHRPPRTIGDKISCYGTLFFRKCFDLVTGYAVPDPDKPDQYKGTRWEMTEEKWMTRCIFLESIAGVPGSVAGFVRHLHSLRMLTRDKAWIETLHDEAYNERMHLLTFIKIGKPSWFTRSIIYIGQGVFTNIFFLVYLMNPRYCHRFVGYLEEEAVRTYTHLIDELDDPNKLPDFQKLPIPNIAVQYWPELTPESSFKDLILRIRADEAKHREINHTFANLEQWQDRNPFALKIKDSDKPQPNYNLDVTRPQGWERKDLYL

>Candida_albicans_AOX0b

MLTASLYKQLPVLTTTATSTYSFIRLSSTLATPPHSTTTTSPSSPAFHQPNHPQQFANPNTSVFDVSTRIYTQEGIDNNNDTKFLTKAPYPHPVFPQDECENVTVTHRETKTLGDKISFRSIQFMRQCFDLVTGYAVPKTNNPDEFKGTRWEMTEGKWLTRCIFLESVAGVPGSVAGFLRHLHSLRMLRRDKAWIETLLDEAYNERMHLLTFIKIGKPSWFTRSIIYVGQGVFTNVFFLLYLLNPRYCHRFVGYLEEEAVRTYSHLLDELAVPGKLPAFETMKIPEVAVQYWPELTPKSSFKDLILRIRADEAKHREVNHTFANLEQKTDRNPFALKIEGLNKPQPNHGINVMRPTGWEK

>Chlamydomonas_reinhardtii_AOX0a

MLQTAPMLPGLGPHLVPQLGALASASRLLGSIASVPPQHGGAGFQAVRGFATGAVSTPAASSPGHKPAATHAPPTRLDLKPGAGSFAAGAVAPHPGINPARMAADSASAAAGASGDAALAESYMAHPAYSDEYVESVRPTHVTPQKLHQHVGLRTIQVFRYLFDKATGYTPTGSMTEAQWLRRMIFLETVAGCPGMVAGMLRHLKSLRSMSRDRGWIHTLLEEAENERMHLITFLQLRQPGPAFRAMVILAQGVFFNAYFIAYLLSPRTCHAFVGFLEEEAVKTYTHALVEIDAGRLWKDTPAPPVAVQYWGLKPGANMRDLILAVRADEACHAHVNHTLSQLNPSTDANPFATGASQLP

>Chlamydomonas_reinhardtii_AOX0b

MTSLPALVPFAALPALVPFAALASTGRLLGSMSGLVCGAQRRLPAHTAFARSHGTATGHAGIVGGAGLSHVKDAAQAFGANQSSSSPSFATSGVAPHPGMKAPSPPTDDEVEACWRPVYDTAYLEKVKPFHITPERLYQRIGFRAIMAARWTFDKLTGYGPNMTEAKWLQRMIFLETIAGVPGMVAGVLRHLKSLRSMKRDHGWIHTLLQEAENERMHLLTFFELRKPGPLFRASIIVAQGVFWNLYFIGYLVSPRTCHAAVGFLEEEAVKTYTHALQEIDAGRLWKGKVAPPIACEYWGLKPGASMRDLILAVRADEACHAHVNHTLSGLPATAPNPFAYGASQLP

>Cicer_arietinum_AOX1

MMMLRQGGMSTTMLLAKKGLVGEVGVPNKWGCLVRAPFVRNESTFASNLPEKQNGSTSSPKSGGNKDEKRVVSYWGIQPSKIVKQDGTEWKWNCFKPWETYKADVTIDLNKHHAPTTFLDKMAYWTVKSLRFPTDLFFQKRYGCRAMMLETVAAVPGMVGGMLLHCKSLRRFEHSGGWIKALLEEAENERMHLMTFMEVAKPKWYERALVITVQGVFFNAYFLGYLISPKFAHRMVGYLEEEAIYSYTEFLKELDKGKIENVPAPAIAIDYWQLPQNSTLRDVVMVIRADEAHHRDVNHFASDIHYQGRELREAAAPIGYH

>Cicer_arietinum_AOX2a

MKHSALCYVVRRALINGRNCNRHGSAVARPLLAAEIRQSGDGGFFYWKRMMSSQAARETEVKKKEKVESSRTEGGKRENNVVVSSYWGISRPKIKREDGTEWPWNCFMPWETYESNLSIDLTKHHAPKNFLDKVAYRTVKLLRIPTDLFFKRQYGCRAMMLETVAAVPGMVGGMLLHLRSLRKFQQSGGWIKALLEEAENERMHLMTMVELVKPKWYERLLVLAVQGVFFNAFFVLYILSPKLAHRVVGYLEEEAIHSYTEYLKDIESGAVENVPAPAIAIDYWRLPKDARLKDVITVIRADEAHHRDVNHFASDIHFHGKELREAPAPLGYH

>Cicer_arietinum_AOX2b

MKHILVRSTVRALFRSGQNYHRNVHRHGGGAFGSFYWRRMSTLPEKKEDHQSDSDENNNKNSNPVVSSYWGITRPKVHREDGTEWPWNCFMPWESYNSDVSIDVTKHHVPTTFGDKFAFRSVKFLRVLSDLYFKERYGCHAMMLETIAAVPGMVGGMLLHLKSLRKFQHTGGWIKALLEEAENERMHLMTMVELVKPSWHERLLVITAQGVFFNAFFVFYLFSPKIAHRFVGYLEEEAVISYTQHLNAIESGKVENVAAPAIAIDYWRLPKDATLKDVIT_VIRADEAHHRDVNHFASDIHHQGKELKEAPAPVGYH

>Citrullus_lanatus_AOX2

MNRIVIRSFVRGLVNSRSFGSVAGVAGGGSGGGMKVTFAAVASPGTRYQPMVDGGFQWRRLLSSSTAVAEEQGKVKLEQGIKDIENQEKKEKETENALVSSYWGIYRPKITREDGSEWPWNCFMPWETYRADLSIDLGKHHEPKTFLDKVAYRVVKLLRIPTDIFFQRRYGCRAVMLETVAAVPGMVGGMLLHLKSLRKFQHSGVWIKALLEEAENERMHLMTMIELVQPKWYERLLVITVQGVFFNAFFVLYLMSPKLAHRIVGYLEEEAIHSYTEYLKDIDEGKIENVPAPAIAIDYWRLPKDARLKDVITVIRADEAHHRDVNHFASDIHFQGKELRESAAPLGYH

>Crassostrea_gigas_AOX

MGSLRQITKLSENGVRIFCSQLKNLENNSILLRVSGIRTSNGLRNAGTKADVDENIKKFKEENFEKIPDPEQLDHFRKTQSTDQLVESMKNPPPMGTHTLPHPIWSEEELHSVKVTHKPPEGFVDKLAFRSVKLLRSTFDLLTGFNWGERTEKKWVLRICFLETVAGVPGMVAAMTRHLHSLRRLKRDHGWIHTLLEEAENERMHLMTALQLRQPSWLFRSGVIVSQGAFVTMFSIAYMLSPRFCHRFVGYLEEEAVFTYSKCLKDIESGSLKHWQTKAAPDVAIRYWKLPETASMKDVVLAIRADEAHHRVVNHTLASMKEDEYNPYEPGK

>Cucumis_melo_AOX2

MNRIVIRSLVRGLVHSRSFSSVAGVPGGARMKVTFAAVASPGTRLQPSADGGFQWRRFLNSSTAVAEEQGKVKSEQGVKDIENPQEKEKEKDNALVSSYWGIYRPKITREDGSEWPWNCFMPWETYRADLSIDLGKHHEPRTFLDKVAYRVVKLLRIPTDIFFQRRYGCRAVMLETVAAVPGMVGGMLLHLKSLRKFQHSGGWIKALLEEAENERMHLMTMIELVQPKWYERLLVITVQGVFFNAFFVLYLMSPKLAHRIVGYLEEEAIHSYTEYLKDINEGKIENVPAPAIAIDYWRLPKDARLKDVITVIRADEAHHRDVNHFASDIHFQGKELRESAAPLGYH

>Cucumis_sativus_AOX2

MNRIVIRSLLRGLLHSRNFGSVAGVPGGARMQLTFAAVASPGTRHQSLSDGGFQWRRFLTSSKAVAEEQGKVKLEQGVKDIENNQEKEKEKDNALVSSYWGIYRPKITREDGSEWPWNCFMPWETYRADLSIDLGKHHQPKTFLDKVAYRVVKLLRIPTDIFFQRRYGCRAVMLETVAAVPGMVGGMLLHLKSLRKFQHSGGWIKALLEEAENERMHLMTMIELVQPKWYERLLVITVQGVFFNAFFVLYLMSPKLAHRIVGYLEEEAIHSYTEYLKDINEGKIENVPAPAIAIDYWRLPKDARLKDVITVIRADEAHHRDVNHFASDIHFQGKELRESAAPLGYH

>Glycine_max_AOX1

MMMMMSRSGANRVANTAMFVAKGLSGEVGGLRALYGGGVRSESTLALSEKEKIEKKVGLSSAGGNKEEKVIVSYWGIQPSKITKKDGTEWKWNCFSPWGTYKADLSIDLEKHMPPTTFLDKMAFWTVKVLRYPTDVFFQRRYGCRAMMLETVAAVPGMVAGMLLHCKSLRRFEHSGGWFKALLEEAENERMHLMTFMEVAKPKWYERALVITVQGVFFNAYFLGYLLSPKFAHRMFGYLEEEAIHSYTEFLKELDKGNIENVPAPAIAIDYWQLPPGSTLRDVVMVVRADEAHHRDVNHFASDIHYQGRELREAAAPIGYH

>Glycine_max_AOX2a

MKLTALNSTVRRALLNGRNQNGNRLGSAALMPYAAAETRLLCAGGANGWFFYWKRTMVSPAEAKVPEKEKEKEKAKAEKSVVESSYWGISRPKVVREDGTEWPWNCFMPWESYRSNVSIDLTKHHVPKNVLDKVAYRTVKLLRIPTDLFFKRRYGCRAMMLETVAAVPGMVGGMLLHLRSLRKFQQSGGWIKALLEEAENERMHLMTMVELVKPKWYERLLVLAVQGVFFNAFFVLYILSPKVAHRIVGYLEEEAIHSYTEYLKDLESGAIENVPAPAIAIDYWRLPKDARLKDVITVIRADEAHHRDVNHFASDIHFQGKELREAPAPIGYH

>Glycine_max_AOX2b

MKNVLVRSAARALLGGGGRSYYRQLSTAAIVEQRHQHGGGAFGSFHLRRMSTLPEVKDQHSEEKKNEVNGTSNAVVTSYWGITRPKVRREDGTEWPWNCFMPWDSYHSDVSIDVTKHHTPKSLTDKVAFRAVKFLRVLSDIYFKERYGCHAMMLETIAAVPGMVGGMLLHLKSLRKFQHSGGWIKALLEEAENERMHLMTMVELVKPSWHERLLIFTAQGVFFNAFFVFYLLSPKAAHRFVGYLEEEAVISYTQHLNAIESGKVENVPAPAIAIDYWRLPKDATLKDVVTVIRADEAHHRDVNHFASDIHHQGKELKEAPAPIGYH

>Lotus_japonicus_AOX1

MMMMRRGVVNTALLAAKGSWVRAPASASSMYGGGLRSFSTAMAEKEVEPSPQHSAGGKKDEKGVMSYWGIQTSKVTKQDGTEWKWNCFRPWETYKADVSIDLEKHHPTKTFMDKLAYWTVKSLRYPTDLFFQRRYGCRAMMLETVAAVPGMVAGMLLHLKSLRRFEHSGGWIKALLEEAENERMHLMTFMEVSDPKWYERALVITVQGVFFNAYFFGYLVSPKFAHRVVGYLEEEAIHSYTEFLKELDSGKIENVPAPAIAIDYWQLPQNATLRDVVMVVRADEAHHRDVNHFASDIILQGRELRDAPAPIGYH

>Lotus_japonicus_AOX2a

MKHLALSYALRRALNCNRHGLTAVRQLPATEVRRFLVSGENGVFSCWNRMMSSQAAPEEEKKEEKAEKESLRTEAKKNDGSVVVSSYWGISRPKITREDGTEWPWNCFMPWETYRPDLSIDLTKHHVPKNFLDKVAYRTVKLLRIPTDVFFQRRYGCRAMMLETVAAVPGMVGGMLLHLRSLRKFQQSGGWIKALLEEAENERMHLMTMVELVQPKWYERFLVLTVQGVFFNAFFVLYLLSPKVAHRVVGYLEEEAIHSYTEYLKDIESGAIENVPAPAIAIDYWRLPKDATLKDVITVIRADEAHHRDVNHFASDIHFHGKELREAPAPLGYH

>Lotus_japonicus_AOX2b

MKHTLARSATRALFNSAARHQHGGGACGSLFWRRMSTLPEVKDHQSEEKKSEVNRNDSSNNTVVPSYWGITRPKVRREDGTEWPWNCFSPWDSYRADVSIDVTKHHLPKTVTDKVAFRAVKFLRVLSDLYFKERYGCHAMMLETIAAVPGMVGGMLLHLKSLRKFQHSGGWIKALLEEAENERMHLMTMTELVKPSWHERLLVITAQGVFFNFFFVFYLLSPKAAHRFVGYLEEEAVISYTDHLNAIERGEVENVPAPAIAIDYWRLPKDATLKDVVTVIRADEAHHRDVNHFASDIHHQGKELKEAPAPVGYH

>Medicago_sativa_AOX1

MMMRHGGHGAAMNTVMLFAKKGLLGGEVGVPNKWGYFVRSTPLVRNASTFTANLSDQKDDKTVDKTPPSSSSQGGAGDNKDEKGITSYWGVQPSKITKPDGTEWKWNCFRPWETYKADVTIDLTKHHKPTTFLDKMAYWTVKSLRYPTDLFFQRRYGCRAMMLETVAAVPGMVGGMLLHCKSLRRFEHSGGWIKALLEEAENERMHLMTFMEVAKPKWYERALVITVQGVFFNAYFLGYLLSPKFAHRMVGYLEEEAIHSYTEFLKELDKGNIENVPAPAIAIDYWQLPQNSTLRDVVEVVRADEAHHRDVNHFASDIHYQGRELREAAAPIGYH

>Medicago_sativa_AOX2a

MKHSALCYMARRALIGGRNSNRQSSAVVRSFAAAEIGQRHLYADGGNGGFFYWKRMMSSQAAPSKPSAEETEAKSTEKNEKKEESSGTKNNVVVSSYWGISRPKIMREDGTEWPWNCFMPWETYQSNVSIDLNKHHVPKNFLDKVAYRTVKLLRIPTDVFFKRRYGCRAMMLETVAAVPGMVGGMLLHLKSLRKFQHSGGWVKALLEEAENERMHLMTMVELVKPKWYERFLVLAVQGVFFNAFFVLYILSPKVAHRVVGYLEEEAIHSYTEYLKDIDSGAIENVPAPAIAIDYWRLPKDAKLKDVITVIRADEAHHRDVNHFASDIHFHGKELRDAPAPLGYH

>Medicago_sativa_AOX2b1

MRNILIRSTARALFRSGGNYHRSFSTAVIVQPRQHQHGGGACGSLYWQRMSTLPEKKDQQTEENKNDAKNNGNNSNAVVSSYWGISRPKVLKEDGTEWPWNCFMPWESYSSDVSIDVTKHHVPKTFGDKLAFRSVKFLRVLSDLYFKERYGCHAMMLETIAAVPGMVGGMLLHLKSLRKFQHAGGWIKALLEEAENERMHLMTMVELVKPSWHERLLVITAQGVFFNGFFVFYILSPKIAHRFVGYLEEEAVISYTQHLDAIESGKVENVPAPAIAIDYWRLPKDATLKDVITVIRADEAHHRDVNHFASDIHHQGKELKEAPAPVGYH

>Medicago_sativa_AOX2b2

MKNLLLRSTARALFCSSQSYHSGLSTAVTVQPRHQNGGGALGSFYWQKMSTLPEKKDQRSEENKNSNDSNTVVSSYWGITRPKVKREDGTEWPWNCFMPWESYSSDVSIDVTKHHVPKTFGDKFAFRSVKFLRVLSDLYFKERYGCHAMMLETIAAVPPMVGGMLLHLKSLRKFQHTGGWIKALLEEAENERMHLMTMVELVKPSWHERLLVITAQGVFFNAFFVFYILSPKTAHRFVGYLEEEAVISYTQHLDAIESGKVENVPAPAIAIDYWRLPKDATLKDVITVIRADEAHHRDVNHFASDIHHQGKELKEAPAPVGYH

>Medicago_truncatula_AOX1

MMMRHGGAMNTAMMFAKKGLLGGEVGVPNKWGYLVRSTPLVRKTSTFTANLSDQKDNKNVDKTPPSSQGGAGDNKDEKGITSYWGVQPSKITKPDGTEWKWNCFRPWETYKADVTIDLTKHHKPTTFLDKMAYWTVKSLRWPTDIFFQRRYGCRAMMLETVAAVPGMVGGMLLHCKSLRRFEQSGGWIKALLEEAENERMHLMTFMEVAKPKWYERALVITVQGVFFNAYFLGYLLSPKFAHRMVGYLEEEAIHSYTEFLKELDKGNIENVPAPAIAIDYWQLPQNSTLRDVVEVVRADEAHHRDVNHFASDIHYQGRELREAAAPIGYH

>Medicago_truncatula_AOX2b1

MRNILLRSTARALFRNGGNYHRSFSTAVIVQPRHHQHGGGACGNLYWQRMSTLPEKKDQQTEESKKDANHNAVVSSYWGISRPKVLKEDGTEWPWNCFMPWESYSSDVSIDVTKHHVPKTFGDKFAFRSVKFLRVLSDLYFKERYGCHAMMLETIAAVPGMVGGMLLHLKSLRKFQHAGGWIKALLEEAENERMHLMTMVELVKPSWHERLLVITAQGVFFNGFFVFYILSPKIAHRFVGYLEEEAVISYTQYLNAIESGKVENVPAPAIAIDYWRLPNDATLKDVVTVIRADEAHHRDVNHFASDIHHQGKELKEAPAPVGYH

>Medicago_truncatula_AOX2b2

MKNSLLRSTARALFHSSRNYHCSFSTAVIVQPRHQNGGGTRGSFYWQKMSTLPEKKDQHSEENKNSNDSNTVVSSYWGITRPKVKREDGTEWPWNCFMPWESYSSDVSIDVTKHHVPKTFGDKFAFRSVKFLRVLSDLYFKERYGCHAMMLETIAAVPPMVGGMLLHLKSLRKFQHTGGWIKALLEEAENERMHLMTMVELVKPSWHERLLVITAQGVFFNAFFVFYILSPKTAHRFVGYLEEEAVISYTQHLNAIESGKVENVPAPAIAIDYWRLPKDATLKDVITVIRADEAHHRDVNHFASDIHHQGKELKEAPAPIGYH

>Medicago_truncatula_AOX2a

MKHSALCYVARRALIGGRNSNRQSSAVVRSFAAAEIGQKHLYADGGNGGLFYWKRMMSSQAAPSKPSAEETEAKSTEKNEKKKEESSGTKNNVVASSYWGISRPKIMREDGTEWPWNCFMPWETYQSNVSIDLNKHHVPKNFLDKVAYRTVKLLRIPTDVFFKRRYGCRAMMLETVAAVPGMVGGMLLHLKSLRKFQHSGGWVKALLEEAENERMHLMTMVELVKPKWYERFLVLAVQGVFFNAFFVLYILSPKVAHRVVGYLEEEAIHSYTEYLKDIDSGAIENVPAPAIAIDYWRLPKDAKLKDVITVIRADEAHHRDVNHFASDIHFHGKELRDAPAPLGYH

>Neurospora_crassa_AOX

MNTPKVNILHAPGQAAQLSRALISTCHTRPLLLAGSRVATSLHPTQTNLSSPSPRNFSTTSVTRLKDFFPAKETAYIRQTPPAWPHHGWTEEEMTSVVPEHRKPETVGDWLAWKLVRICRWATDIATGIRPEQQVDKHHPTTATSADKPLTEAQWLVRFIFLESIAGVPGMVAGMLRHLHSLRRLKRDNGWIETLLEESYNERMHLLTFMKMCEPGLLMKTLILGAQGVFFNAMFLSYLISPKITHRFVGYLEEEAVHTYTRCIREIEEGHLPKWSDEKFEIPEMAVRYWRMPEGKRTMKDLIHYIRADEAVHRGVNHTLSNLDQKEDPNPFVSDYKEGEGGRRPVNPALKPTGFERAEVIG

>Novosphingobium_aromaticivorans_DSM_12444_AOX

MIPPFIDLSVHHKPGGLSDRIAFGFTKALRWCADTFFAERYGHRAVVLETVAAVPGMVGATINHLACLRRMCDDKGWIKTLMDEAENERMHLMTFIEISKPTLFERAVIMGVQWVFYLFFFGLYLVSPKTAHRVVGYFEEEAVISYTHYLAEIDQGRSANVPAPAIAKRYWGLPDNAMLRDVVLVVRADEAHHRDVNHGFANELAGLPVAEPAACPPHHALEPNWKKAA

>Phaseolus_vulgaris_AOX1

MMMMSRGGTKRVANTAVSVAKGLSCEVGGLKAFYGGGVRSESTMALSEKEKKVGLSSDGGNKEQKEIVSYWGVEPSKITKLDGTEWKWNCFRPWETYKADVSIDLKKHHPPTTFLDKMAFWTVKTLRYPTDVFFQRQYGCRAMMLETVAAVPGMVGGMLLHFKSLRRFEQSGGWIKALLEEAENERMHLMTFMEVAKPKWYERALVITVQGVFFNAYFLGYMISPKFAHRMVGYLEEEAIHSYTEFLKELDKGNIQNVPAPAIAIDYWQLPPDATLRDVVMVVRADEAHHRDVNHFASDIHYQGRELRETAAPIGYH

>Phaseolus_vulgaris_AOX2a

MKFIAFSYTVRRALLNGRNCNHLGSTAVMACAAPETRLLCAGAANGGLFYWRRSMASPAEAKLPEKDKEKEKAEKSVVESSYWGISRPRIMREDGTEWPWNCFMPWETYQSNLSIDLTKHHVPKNFPDKVAYRTVKLLRIPTDLFFQRRYGCRAMMLETVAAVPGMVGGMLLHLRSLRKFQQSGGWIKALMEEAENERMHLMTMVELVKPKWYERLLVLAVQGVFFNAFFALYILSPKVAHRIVGYLEEEAIHSYTEYLKDIERGAIENVPAPAIAIDYWRLPKDAKLKDVITVIRADEAHHRDVNHFASDIHFQGKELREAPAPLGYH

>Phaseolus_vulgaris_AOX2b

MKHTLVKSAAQALIGGGRSYYRHLPTAKILEPTHQHGGGAFGSFYLRRMSTLPDTKDHNSEENKNEVKENNTNAVTSSYWGISRPKVHREDGTEWPWNCFMPWDTYHSDVSIDVTKHHTPKSLTDKVAFKSVKFLRVLSDLYFKERYGCHAMMLETIAAVPGMVGGMLLHLKSLRKFQHSGGWIKALLEEAENERMHLMTMVELVNPKWHERLLIFAAQGVFFNGFFVFYLLSPKAAHRFVGYLEEEAVISYTQHLEAIESGKVENVPAPAIAIDYWRLPKDATLKDVVTVIRADEAHHRDVNHFASDIHHQGKELRDAPAPVGYH

>Trypanosoma_brucei_brucei_AOX

MFRNHASRITAAAAPWVLRTACRQKSDAKTPVWGHTQLNRLSFLETVPVVPLRVSDESSEDRPTWSLPDIENVAITHKKPNGLVDTLAYRSVRTCRWLFDTFSLYRFGSITESKVISRCLFLETVAGVPGMVGGMLRHLSSLRYMTRDKGWINTLLVEAENERMHLMTFIELRQPGLPLRVSIIITQAIMYLFLLVAYVISPRFVHRFVGYLEEEAVITYTGVMRAIDEGRLRPTKNDVPEVARVYWNLSKNATFRDLINVIRADEAEHRVVNHTFADMHEKRLQNSVNPFVVLKKNPEEMYSNQPSGKTRTDFGSEGAKTASNVNKHV

>Vigna_unguiculata_AOX1

MMMSRSGGNRVANAVMLVAKGLSGEVGGARAFYGGGVRSESTLVLPEKEKMEKKVGDGGNKEQKGIVSYWGVEPSKITKLDGTEWKWNCFRPWETYKADVSIDLNKHHAPTTFLDKMALWTVKTLRYPTDLFFQRRYGCRAMMLETVAAVPGMVAGMLLHLKSLRRFEHSGGWIKALLEEAENERMHLMTFMEVAKPKWYERALVITVQGVFFNAYFLGYMISPKFAHRMVGYLEEEAIHSYTEFLKELDKGNIENVPAPAIAIDYWQLPPDSTLKDVVTVVRADEAHHRDVNHFASDIHYQGRELREAAAPIGYH

>Vigna_unguiculata_AOX2a

MKFIALSCTVRRALLNGRNCNGLGSTAVMAYAAPETRFLCAGAANGGLFYWRRSMASQAEAKLPEKDKEKAEAEKSVVESSYWGISRPRIMREDGTEWPWNCFMPWETYHSNLSIDLTKHHVPKNFLDKVAYRTVKLLRIPTDLFFQRRYGCRAMMLETVAAVPGMVGGMLLHLRSLRKFQQSGGWIKALMEEAENERMHLMTMVELVKPKWYERLLVIAVQGVFFNAFFVLYILSPKVAHRIVGYLEEEAIHSYTEYLKDIESGAIENVPAPAIAIDYWRLPKDAKLKDVITVIRADEAHHRDVNHFASDIHFQGKELREAPAPIGYH

>Vigna_unguiculata_AOX2b

MKHTLVRSAARALLGGGRSYYRHAPTAAIVEPTRQHGGGAFGSFYLRRMSTLPDIKDHNSEEKKNEVKDDNTNAVISSYWGISRPKVRREDGTEWPWNCFMPWDTYHSDVSIDVTKHHTPKSLTDKVAFRSVKFLRVLSDLYFKERYGCHAMMLETIAAVPGMVGGMLLHLKSLRKFQHSGGWIKALLEEAENERMHLMTMVELVQPKWHERLLIFTAQGVFFNAFFVFYLLSPKAAHRFVGYLEEEAVISYTQHLEAIESGKVENVPAPAIAIDYWRLPKDATLKDVVTVIRADEAHHRDVNHFASDIHHQGKELRDAPAPIGYH
