# Supplementary material for: Cucumber Possesses a Single Terminal Alternative Oxidase Gene That is Upregulated by Cold Stress and in the Mosaic (MSC) Mitochondrial Mutants
Source: Plant Mol Biol Report. 2015 Apr 21;33:1893–906. doi: 10.1007/s11105-015-0883-9 (PMC4695503; doi:10.1007/s11105-015-0883-9)
Supplement: Supplementary file 2 — Amino acids sites specific to AOX2 in cucumber, melon, and watermelon. An alignment of the conservative fragment (100–354 aa) of AOX2a–c consensus sequence (Costa et al. 2014) and cucumber, melon, and watermelon corresponding AOX fragments. (DOCX 41 kb) [file 11105_2015_883_MOESM2_ESM.docx]

**Cucumber possesses a single terminal alternative oxidase gene that is upregulated by cold stress and in the mosaic (MSC) mitochondrial mutants**

Journal: Plant Molecular Biology Reporter

Authors: Tomasz L. Mróz^A^, Michael J. Havey^B^, Grzegorz Bartoszewski^*A^

^A^Department of Plant Genetics, Breeding and Biotechnology, Faculty of Horticulture, Biotechnology and Landscape Architecture, Warsaw University of Life Sciences, ul. Nowoursynowska 159, 02-776 Warsaw, Poland

^B^Agricultural Research Service, U.S. Department of Agriculture, Vegetable Crops Unit, Deptartment of Horticulture, 1575 Linden Dr., University of Wisconsin, Madison, WI 53706, USA

*email: grzegorz_bartoszewski@sggw.pl

**Supplementary file 2** Amino acids sites specific to AOX2 in cucumber, melon, and watermelon.

**AOX2a-c** .**vvvsSYWG! srpk!tr#DG teWPWNCFMP W#tY.a#lsI DL.KHHvPkt 150**

*C. sativus*  .A**L**VSSYWGI **Y**RPKITREDG SEWPWNCFMP WETYRADLSI DLGKHH**Q**PKT 150

*C. melo*  .A**L**VSSYWGI **Y**RPKITREDG SEWPWNCFMP WETYRADLSI DLGKHH**E**PRT 150

*C. lanatus*  .A**L**VSSYWGI **Y**RPKITREDG SEWPWNCFMP WETYRADLSI DLGKHH**E**PKT 150

*****

v

**AOX2a-c FlDKvA%rTV KlLRiPTDiF FqRRYGCRAM MLETVAAVPG MVGGMLLHLr 200**

*C. sativus* FLDKVAYR**V**V KLLRIPTDIF FQRRYGCRA**V** MLETVAAVPG MVGGMLLHLK 200

*C. melo* FLDKVAYR**V**V KLLRIPTDIF FQRRYGCRA**V** MLETVAAVPG MVGGMLLHLK 200

*C. lanatus*  FLDKVAYR**V**V KLLRIPTDIF FQRRYGCRA**V** MLETVAAVPG MVGGMLLHLK 200

*****

I

**AOX2a-c SLRkF#qSGG WIKALLEEAE NERMHLMTMV ELVkPkWYER lLVLtVQGVF 250**

*C. sativus* SLRKFQHSGG WIKALLEEAE NERMHLMTM**I** ELVQPKWYER LLVITVQGVF 250

*C. melo*  SLRKFQHSGG WIKALLEEAE NERMHLMTM**I** ELVQPKWYER LLVITVQGVF 250

*C. lanatus* SLRKFQHSGV WIKALLEEAE NERMHLMTM**I** ELVQPKWYER LLVITVQGVF 250

*****

**AOX2a-c FNa%FvlY.l SPKlAHR!VG YLEEEA!HSY TE%LKDI#sG aI#NVpAPAI 300**

*C. sativus* FNAFFVLYLM SPKLAHRIVG YLEEEAIHSY TEYLKDIN**E**G KIENVPAPAI 300

*C. melo* FNAFFVLYLM SPKLAHRIVG YLEEEAIHSY TEYLKDIN**E**G KIENVPAPAI 300

*C. lanatus* FNAFFVLYLM SPKLAHRIVG YLEEEAIHSY TEYLKDID**E**G KIENVPAPAI 300

**AOX2a-c AIDYWRLPKD atLkDV!tVI RADEAHHRDV NHFASDIH%# GKeLr#apAP 350**

*C. sativus* AIDYWRLPKD ARLKDVITVI RADEAHHRDV NHFASDIHFQ GKELRE**S**AAP 350

*C. melo*  AIDYWRLPKD ARLKDVITVI RADEAHHRDV NHFASDIHFQ GKELRE**S**AAP 350

*C. lanatus* AIDYWRLPKD ARLKDVITVI RADEAHHRDV NHFASDIHFQ GKELRE**S**AAP 350

*****

**AOX2a-c lGYH 354**

*C. sativus* LGYH 354

*C. melo*  LGYH 354

*C. lanatus* LGYH 354

An alignment of the conservative fragment (101-354 aa) of AOX2a–c consensus sequence (Costa et al. 2014) and cucumber, melon, and watermelon corresponding AOX fragments appears with 16 specific amino acid residues (marked in purple). Amino acids sites marked in purple and highlighted in white font were rated as exhibiting relevant differences using the ‘sequence harmony’ (SH) methodology (Feenstra et al. 2007). In addition, those presenting the strong reliability parameters in SH ≤ 0.13 are marked with an asterisk (*). Yellow selection in AOX2 consensus sequence represents residues specific to AOX2a–c subtype and green selection above AOX2 consensus sequence represents residues specific to AOX1d (Costa et al. 2014) for which there is compliance with the AOX2 sequences of tested cucurbits (described in detail in the manuscript). Figure was prepared by using a model proposed by Costa et al. (2014). Four highly conserved AOX active regions (LET, NERMHL, LEEEA, and RADE__H) are marked by frames, glutamic acid (E) and histidine (H) amino acid residues involved in iron-binding are indicated by black circles (Berthold et al. 2000). Capital letters at the specific sites represent the most frequently seen amino acids. Consensus symbols used in the AOX2a–c consensus sequences: ! is I (Isoleucine), or V (valine); % is F (phenylalanine), or Y (tyrosine); # is N (asparagine), D (aspartic acid), Q (glutamine), or E (glutamic acid).
