# Supplementary material for: Cucumber Possesses a Single Terminal Alternative Oxidase Gene That is Upregulated by Cold Stress and in the Mosaic (MSC) Mitochondrial Mutants
Source: Plant Mol Biol Report. 2015 Apr 21;33:1893–906. doi: 10.1007/s11105-015-0883-9 (PMC4695503; doi:10.1007/s11105-015-0883-9)
Supplement: Supplementary file 3 — Description and primer sequences of candidate genes tested as potential RT-qPCR references for cucumber line B (wild-type) and MSC mutants grown under optimal and cold stress growth conditions. (DOCX 19 kb) [file 11105_2015_883_MOESM3_ESM.docx]

**Cucumber possesses a single terminal alternative oxidase gene that is upregulated by cold stress and in the mosaic (MSC) mitochondrial mutants**

Journal: Plant Molecular Biology Reporter

Authors: Tomasz L. Mróz^A^, Michael J. Havey^B^, Grzegorz Bartoszewski^*A^

^A^Department of Plant Genetics, Breeding and Biotechnology, Faculty of Horticulture, Biotechnology and Landscape Architecture, Warsaw University of Life Sciences, ul. Nowoursynowska 159, 02-776 Warsaw, Poland

^B^Agricultural Research Service, U.S. Department of Agriculture, Vegetable Crops Unit, Department of Horticulture, 1575 Linden Dr., University of Wisconsin, Madison, WI 53706, USA

*email: grzegorz_bartoszewski@sggw.pl

**Supplemental table S1** Description and primer sequences of 13 candidate genes tested as potential RT-qPCR reference genes for cucumber line B (wild-type) and MSC mutants grown under optimal conditions (10 genes tested) and cold stress growth conditions (all 13 genes tested).

| **Abbreviation** | **Gene name** | **Primer sequence (5’ - 3’)** | **Function** | **Orgin** |
| --- | --- | --- | --- | --- |
| *ATP* | ATPase subunit III | concealed by the manufacturer | subunit III of ATPase | Cucumber geNorm Kit (PrimerDesign Ltd) |
| *CACS* | Clathrin adaptor complex subunit | TGGGAAGATTCTTATGAAGTGC | intracellular protein transport, vesicle mediated transport | Migocka and Papierniak (2010) |
|  |  | CTCGTCAAATTTACACATTGGT |  |  |
| *EF*α | Elongation factor 1-alpha | ACTTTATCAAGAACATGATTAC | translational elongation | Migocka and Papierniak (2010) |
|  |  | TTCCTTCACAATTTCATCG |  |  |
| *F-box* | F-box protein/ galactose oxidase/ kelch repeat protein | GGTTCATCTGGTGGTCTT | unknown | Migocka and Papierniak (2010) |
|  |  | CTTTAAACGAACGGTCAGTCC |  |  |
| *GRI* | glutamyl-tRNA reductase, isozyme 1 | concealed by the manufacturer | tetrapyrroles biosynthesis, activation of the glutamine residues into the ribosomal protein biosynthesis, reduction glutamate in plastids | Cucumber geNorm Kit (PrimerDesign Ltd) |
| *M2** | Cyclophilin | concealed by the manufacturer | protein folding, signal transduction | Cucumber geNorm Kit (PrimerDesign Ltd) |
| *mdhG** | Glyoxysomal malate dehydrogenase | concealed by the manufacturer | enzyme of the glyoxylate cycle and tricarboxylic acid cycle, participates in degradation of storage oil | Cucumber geNorm Kit (PrimerDesign Ltd) |
| *NADPH** | Protochlorophyllide oxidoreductase | concealed by the manufacturer | catalysis of light-dependent reduction of protochlorophyllide | Cucumber geNorm Kit (PrimerDesign Ltd) |
| *PLD* | Phospholipase D | concealed by the manufacturer | phosphatidic acid production (PA), signal transduction | Cucumber geNorm Kit (PrimerDesign Ltd) |
| *TIP41* | TIP41-like family  protein | CAACAGGTGATATTGGATTATGATTATAC | PP2A phosphatase activator | Migocka and Papierniak (2010) |
|  |  | GCCAGCTCATCCTCATATAAG |  |  |
| *TUA* | α - tubulin | ACGCTGTTGGTGGTGGTAC | structural constituent of cytoskeleton, protein folding | Wan et al. (2010) |
|  |  | GAGAGGGGTAAACAGTGAATC |  |  |
| *TUB* | α - tubulin | CCTCGACATTGAGCGACCTAAC | structural constituent of cytoskeleton, protein folding | Witkowicz et al. (unpublished) |
|  |  | CATCCACGTTCAATGCACCA |  |  |
| *UBI-ep* | Ubiquitin extension protein | CACCAAGCCCAAGAAGATC | protein binding, protein modification | Wan et al. (2010) |
|  |  | TAAACCTAATCACCACCAGC |  |  |

*Reference candidate genes not tested for optimal growth conditions

References

Migocka M, Papierniak A (2011) Identification of suitable reference genes for studying gene expression in cucumber plants

subjected to abiotic stress and growth regulators. Mol Breed 28:343–357

Wan H, Zhao Z, Qian C, Sui Y, Malik AA, Chen J (2010) Selection of appropriate reference genes for gene expression studies by

quantitative real-time polymerase chain reaction in cucumber. Anal Biochem 15:257–261
