# Supplementary material for: Cucumber Possesses a Single Terminal Alternative Oxidase Gene That is Upregulated by Cold Stress and in the Mosaic (MSC) Mitochondrial Mutants
Source: Plant Mol Biol Report. 2015 Apr 21;33:1893–906. doi: 10.1007/s11105-015-0883-9 (PMC4695503; doi:10.1007/s11105-015-0883-9)
Supplement: Supplementary file 4 — Average gene expression stability values (M) of 13 tested RT-qPCR reference candidate genes in the study of AOX2 expression in (a) cucumber MSC mutants grown in the optimal phytotron conditions and (b) cold-treated line B. (DOCX 18 kb) [file 11105_2015_883_MOESM4_ESM.docx]

**Cucumber possesses a single terminal alternative oxidase gene that is upregulated by cold stress and in the mosaic (MSC) mitochondrial mutants**

Journal: Plant Molecular Biology Reporter

Authors: Tomasz L. Mróz^A^, Michael J. Havey^B^, Grzegorz Bartoszewski^*A^

^A^Department of Plant Genetics, Breeding and Biotechnology, Faculty of Horticulture, Biotechnology and Landscape Architecture, Warsaw University of Life Sciences, ul. Nowoursynowska 159, 02-776 Warsaw, Poland

^B^Agricultural Research Service, U.S. Department of Agriculture, Vegetable Crops Unit, Department of Horticulture, 1575 Linden Dr., University of Wisconsin, Madison, WI 53706, USA

*email: grzegorz_bartoszewski@sggw.pl

**Supplemental table S2** Average gene expression stability values (*M*) of tested RT-qPCR candidate reference genes to study *AOX2* expression in (a) cucumber MSC mutants grown in the optimal phytotron conditions and (b) cold treated line B. Gene expression stability was estimated using geNorm V3.4 (Vandesompele et al. 2002). Higher *M*-values indicate less stable genes, while lower *M*-values indicate most stable genes.

1. ***Optimal growth conditions (for line B and MSC mutants)***

| **Rank** | **1** | **1** | **2** | **3** | **4** | **5** | **6** | **7** | **8** | **9** |  |  |  |
| --- | --- | --- | --- | --- | --- | --- | --- | --- | --- | --- | --- | --- | --- |
| **Gene** | *UBI-ep* | *TIP41* | *ATP* | *CACS* | *F-box* | *TUA* | *PLD* | *EF*α | *TUB* | *GRI* |  |  |  |
| **Stability** | 0.197 | 0.197 | 0.231 | 0.286 | 0.307 | 0.321 | 0.347 | 0.499 | 1.774 | 3.020 |  |  |  |
|  | | | | | | | | | | |  |  |  |
| 1. ***Cold stress growth conditions (for line B)*** | | | | | | | | | | | | | |
| **Rank** | **1** | **1** | **2** | **3** | **4** | **5** | **6** | **7** | **8** | **9** | **10** | **11** | **12** |
| **Gene** | *M2* | *mdhG* | *UBI-ep* | *ATP* | *GRI* | *NADPH* | *F-box* | *CACS* | *TUA* | *EF*α | *PLD* | *TUB* | *TIP41* |
| **Stability** | 0.486 | 0.486 | 0.578 | 0.725 | 0.767 | 0.851 | 0.921 | 0.968 | 1.016 | 1.043 | 1.188 | 2.050 | 2.767 |
