# Supplementary material for: Cucumber Possesses a Single Terminal Alternative Oxidase Gene That is Upregulated by Cold Stress and in the Mosaic (MSC) Mitochondrial Mutants
Source: Plant Mol Biol Report. 2015 Apr 21;33:1893–906. doi: 10.1007/s11105-015-0883-9 (PMC4695503; doi:10.1007/s11105-015-0883-9)
Supplement: Supplementary file 5 — Specific amino acids of AOX2a–c and cucurbits AOX protein (C. sativus, C. melo, and C. lanatus) groups detected through the sequence harmony program (SeqHarm version 1.1) using a cutoff 0.2. (DOCX 29 kb) [file 11105_2015_883_MOESM5_ESM.docx]

**Cucumber possesses a single terminal alternative oxidase gene that is upregulated by cold stress and in the mosaic (MSC) mitochondrial mutants**

Journal: Plant Molecular Biology Reporter

Authors: Tomasz L. Mróz^A^, Michael J. Havey^B^, Grzegorz Bartoszewski^*A^

^A^Department of Plant Genetics, Breeding and Biotechnology, Faculty of Horticulture, Biotechnology and Landscape Architecture, Warsaw University of Life Sciences, ul. Nowoursynowska 159, 02-776 Warsaw, Poland

^B^Agricultural Research Service, U.S. Department of Agriculture, Vegetable Crops Unit, Department of Horticulture, 1575 Linden Dr., University of Wisconsin, Madison, WI 53706, USA

*email: grzegorz_bartoszewski@sggw.pl

**Supplemental table S3** Specific amino acids of AOX2a–c consensus and cucurbits AOX proteins (*C. sativus*, *C. melo,* and *C. lanatus)* groups detected through the sequence harmony program (SeqHarm version 1.1) using a cutoff 0.2.

| **Pos** | **Entropy** | | | | **SH** | **Rnk** | **Consensus** | |
| --- | --- | --- | --- | --- | --- | --- | --- | --- |
| **Ali** | **A** | **B** | **AB** | **rel.** |  |  | AOX2a–c | cucurbits AOX proteins |
| 147 | 1.18 | 0.92 | 1.36 | 1.92 | 0.10 | 1 | Vkaelmq | Eq |
| 230 | 0.30 | 0.00 | 0.48 | 1.92 | 0.10 | 1 | Vim | I |
| 159 | 0.50 | 0.00 | 0.66 | 1.92 | 0.10 | 1 | Tivg | V |
| 347 | 0.49 | 0.00 | 0.63 | 1.78 | 0.13 | 1 | Asgt | S |
| 180 | 0.33 | 0.00 | 0.47 | 1.67 | 0.17 | 1 | Mv | V |
| 289 | 1.41 | 0.00 | 1.50 | 1.67 | 0.17 | 1 | Snredt | E |
| 111 | 1.66 | 0.00 | 1.74 | 1.67 | 0.17 | 1 | Sqyapemtv | Y |
| 103 | 2.23 | 0.00 | 2.30 | 1.67 | 0.17 | 1 | Vmalsteq | L |

The most reliable sites are shown from top to bottom denoted by the lowest SH value. Classification was based on the highest rank (Rnk) values and entropy according ‘sequence harmony’ (SH) methodology. Positions in the alignment (Pos Ali) of the conservative fragment (101-354 aa) of AOX2a–c consensus sequence represents the amino acid number in the sequence of the *A. thaliana* AOX1a used as reference model by Costa et al. (2014). Capital letters in the consensus represent the most frequently present amino acids. A decrease of red color intensity represents an increase in SH values:

| 0.10 | 0.12 | 0.14 | 0.16 | 0.18 | 0.20 |
| --- | --- | --- | --- | --- | --- |
